# Overexpression of Na^+^/Mg^2+^ exchanger SLC41A1 attenuates pro-survival signaling

**DOI:** 10.18632/oncotarget.23598

**Published:** 2017-12-22

**Authors:** Gerhard Sponder, Nasrin Abdulhanan, Nadine Fröhlich, Lucia Mastrototaro, Jörg R. Aschenbach, Monika Röntgen, Ivana Pilchova, Michal Cibulka, Peter Racay, Martin Kolisek

**Affiliations:** ^1^ Institute of Veterinary-Physiology, Free University of Berlin, Berlin, Germany; ^2^ PerkinElmer Life and Analytical Sciences GmbH, Rodgau, Germany; ^3^ Leibnitz Institute for Farm Animal Biology, Department of Muscle and Growth Physiology, Dummerstorf, Germany; ^4^ Biomedical Center Martin, Division of Neurosciences, Jessenius Faculty of Medicine in Martin, Comenius University in Bratislava, Martin, Slovakia; ^5^ Institute of Medical Biochemistry, Jessenius Faculty of Medicine in Martin, Comenius University in Bratislava, Martin, Slovakia

**Keywords:** Na^+^/Mg^2+^ exchanger, Mg^2+^ homeostasis, Akt/PKB, dynamic mass redistribution, signaling

## Abstract

The Na^+^/Mg^2+^ exchanger SLC41A1 (A1), a key component of intracellular Mg homeostasis (IMH), is the major cellular Mg^2+^ efflux system, and its overexpression decreases [Mg^2+^]_intracellular_. IMH plays an important role in the regulation of many cellular processes, including cellular signaling. However, whether the overexpression of A1 and the consequent drop of [Mg^2+^]_i_ impact on intracellular signaling is unknown.

To examine the latter, we utilized dynamic mass redistribution (DMR) assay, PathScan^®^ RTK signaling antibody (PRSA) array, confirmatory Western blot (WB) analyses of phosphorylation of kinases selected by PRSA, and mag-fura 2-assisted fast filter spectrometry (FFS).

We demonstrate here that the overexpression of A1 quantitatively and qualitatively changes the DMR signal evoked by the application of PAR-1-selective activating peptide and/or by changing [Mg^2+^]_extracellular_ in HEK293 cells. PRSA profiling of the phosphorylation of important signaling nodes followed by confirmatory WB has revealed that, in HEK293 cells, A1 overexpression significantly attenuates the phosphorylation of Akt/PKB on Thr^308^ and/or Ser^473^ and of Erk1/2 on Thr^202^/Tyr^204^ in the presence of 0 or 1 mM (physiological) Mg^2+^ in the bath solution. The latter is also true for SH-SY5Y and HeLa cells. Overexpression of A1 in HEK293 cells significantly lowers [Mg^2+^]_i_ in the presence of [Mg^2+^]_e_ = 0 or 1 mM. This correlates with the observed attenuation of prosurvival Akt/PKB – Erk1/2 signaling in these cells.

Thus, A1 expression status and [Mg^2+^]_e_ (and consequently also [Mg^2+^]_i_) modulate the complex physiological fingerprint of the cell and influence the activity of kinases involved in anti-apoptotic and, hence, pro-survival events in cells.

## INTRODUCTION

Magnesium (Mg^2+^) has been implicated in numerous vital cellular processes [1, 2, 3]. Its deficiency can have detrimental effects on the life of the cell [1]. Intracellular Mg^2+^ deficiency (IMD) can be caused: (1) by an insufficient supply of the ion from the extracellular milieu; (2) by mutation, i.e., non-functional or faulty cellular Mg^2+^ transport systems; or (3) by disrupted mechanisms regulating the influx, deposition, reposition, and efflux of the ion from the cell [1]. Little is known about the regulation of intracellular magnesium homeostasis (IMH), largely because of the lack of knowledge about the identities of cellular Mg^2+^ transport mechanisms, most of which have been discovered only recently.

Solute carrier SLC41A1 (further referred to as A1) has been characterized as being a Na^+^/Mg^2+^ exchanger (NME) and the major cellular Mg^2+^ efflux system ubiquitously expressed in various cell types [4–7 and http://www.proteinatlas.org/ENSG00000133065-SLC41A1/tissue]. The increased activity of NME is associated with many maladies, e.g., hypertension, preeclampsia, neurodegenerative disorders, cystic fibrosis, and *diabetes mellitus* [6, 8–13]. Even though IMD is assumed to contribute to the clinical image of the aforementioned maladies, whether it is also one of the primary causes of these diseases remains uncertain.

**Figure 1:**
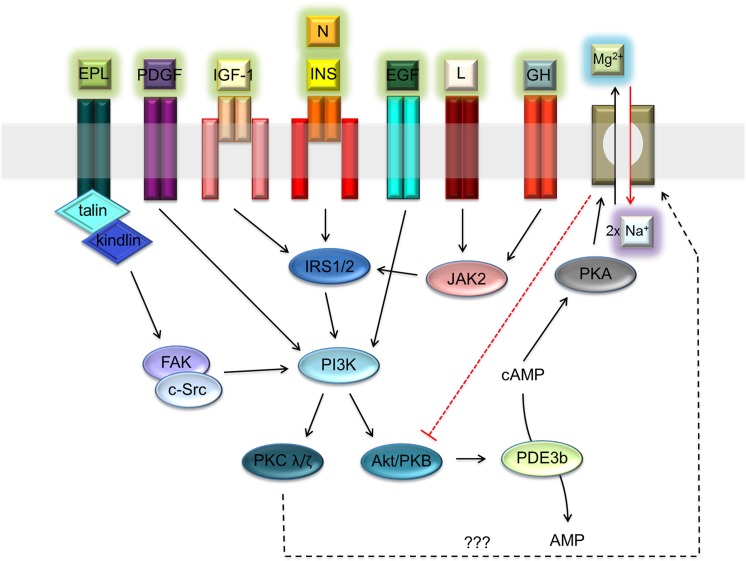
Receptor-ligand network activating PI3K – Akt/PKB signaling node Abbreviations: Akt/PKB, protein kinase B; cAMP, cyclic adenosine monophosphate; c-Src, proto-oncogene tyrosine-protein kinase; EGF, epidermal growth factor; EPL, extracellular polyvalent ligands; FAK, focal adhesion kinase; GH, growth hormone; INS, insulin; IRS1/2, insulin receptor substrate 1 and 2; I, integrin; PDGF, platelet-derived growth factor; IGF-1, insulin-like growth factor 1; JAK2, Janus kinase 2; L, leptin; N, neuritin; PDE3b, phosphodiesterase 3b; PI3K, phosphatidylinositol-4,5-bisphosphate 3-kinase; PKA, protein kinase A; PKC λ/ζ, protein kinase C λ or ζ; R, receptor. Dashed black arrow indicates a speculative link between PKC and Na^+^/Mg^2+^ exchanger [65, 66]. Dashed red line indicates the inhibitory effect of SLC41A1 on Akt/PKB activity.

The function of A1 is regulated by cAMP-dependent protein kinase A (PKA) [5–7]. The increased PKA-dependent phosphorylation of SLC41A1 leads to an increase of Mg^2+^ efflux capacity in transgenic HEK293 cells [5, 6]. Levels of intracellular cAMP are controlled by various hormonal stimuli [13]. In several reports, the authors have demonstrated either the inhibitory (e.g., insulin; INS) or stimulatory (e.g., angiotensin II; ANG) effects of hormones on NME performance [13–15]. In particular, the inhibitory effect of INS may play a protective role against the excessive loss of Mg^2+^ from cells. The INS signaling axis IRTK – PI3K – Akt/PKB with the end effector phosphodiesterase 3b (PDE3b) is assumed to regulate (decrease) the level of cAMP and consequently also of PKA-dependent SLC41A1 activation [13]. Many other extracellular signals might influence the activity of the PI3K-Akt/PKB signaling node. Among these are neuritin signaling via IRTK – PI3K – Akt/PKB; platelet-derived growth factor (PDGF) signaling via PDGFR – PI3K – Akt/PKB; epidermal growth factor (EGF) signaling via EGFR – PI3K – Akt/PKB; insulin-like growth factor 1 (IGF-1) signaling via IGF-1R – PI3K – Akt/PKB; leptin (L) signaling via the LR – JAK2 – IRS2 signaling switch; growth hormone (GH), interferon-gamma (INFγ), and leukemia inhibitory factor (LIF) signaling via the GHR/INFγR/LIFR – JAK2 – IRS1 signaling switch; and extracellular polyvalent-ligand-activating integrin-linked FAK/c-Src dual kinase - PI3K - Akt/PKB signaling (Figure 1) [16–23]. Therefore, a reasonable assumption is that the activity of SLC41A1 in various tissues is regulated by the interplay of various extracellular signals translated into the activity of the PI3K-Akt/PKB signaling node.

In our previous work, we have demonstrated that increased Mg^2+^ efflux capacity is achieved by the overexpression of A1 in HEK293 cells [4, 5]. The overexpression of A1 is also disease-related. Recently, this has been correlated with preeclampsia, a life-threatening condition in pregnant women [10]. Promotor and/or other regulatory sequences of *A1* are assumed to possess androgen-responsive elements (*ARE*s) that bind androgen receptor (AR) dimers [24, 25]. AR dimers then bind other transcription factors and regulate the transcription activity of the gene [11]. Apart from this, nothing is known about the regulation of A1 expression or about any impacts of A1 expression on the complex physiology of the cell.

Here, we have examined the effects of the A1 expression status on dynamic mass redistribution (DMR) signals reflecting an integrated physiological response of the cell to (1) SFLLR-NH_2_ stimulation and (2) the modulation of [Mg^2+^]_e_. Furthermore, we have examined the impact of A1 overexpression and of [Mg^2+^]_e_ on the phosphorylation of receptor tyrosine kinases (RTK) and important cellular signaling nodes.

## RESULTS

### Overexpression of A1 modulates the DMR profile of HEK293 cells

The DMR signal is an integrated response that consists of contributions from many cellular events induced by the ligand, thus providing alternative means for studying cell systems biology [26]. To date, DMR signals have been prevalently studied with regard to three classes of receptors: EGFR, G_q_-coupled receptors, and G_s_-coupled receptors [26]. Here, we have tested the effect of SFLLR-NH_2_ in uninduced cells (further referred to as –tet cells) and tet-induced cells overexpressing A1 (further referred to as +tet cells). SFLLR-NH_2_ is an agonist of G-protein-coupled protease-activated receptor 1 (PAR-1) and, at higher concentrations, also of PAR-2, which is used as a positive DMR control in HEK293 cells (PerkinElmer) [27–29].

**Figure 2:**
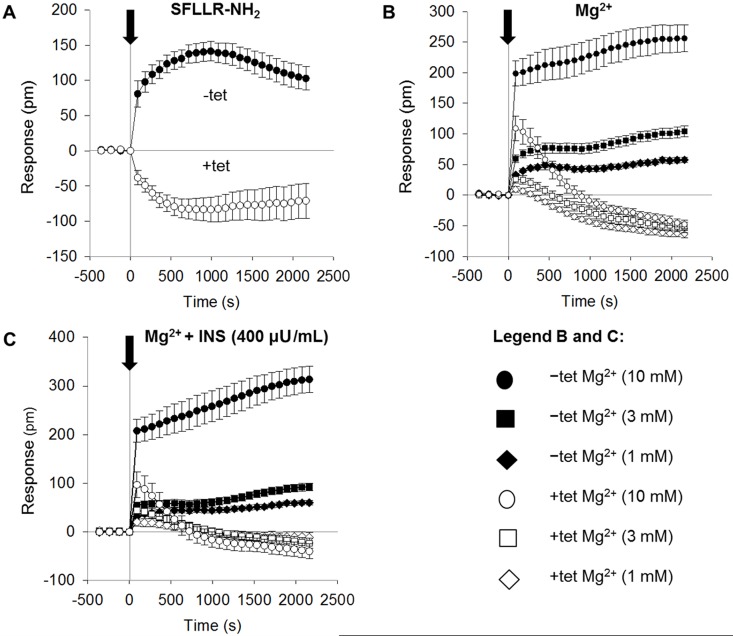
Real-time DMR signals measured in –tet and +tet HEK293 cells and induced by application of **(A)** SFLLR-NH_2_ (5 μM) or **(B)** Mg^2+^ at various concentrations (1, 3, or 10 mM). **(C)** Real-time DMR signals measured in –tet and +tet HEK293 cells and induced by application of Mg^2+^ at various concentrations (1, 3, or 10 mM) in the presence of INS (400 μU/mL) in the examined (CDF-HBSS+) solution. Black arrow indicates the application of SFLLR-NH_2_, of Mg^2+^, or of Mg^2+^ with INS (Time 0 s). Before application of the respective effectors, –tet and +tet cells were equilibrated in CMF-HBSS+ for 90 min (60 min equilibration plus 30 min baseline measurements). DMR signals are given as the response in picometers (pm). Data were acquired at 90 s intervals. Data are presented as means (N = 15 for each tested condition) ± SE. Abbreviations: DMR, dynamic mass redistribution; INS, insulin; SFLLR-NH_2_, protease-activated receptor 1 activating peptide; tet, tetracycline.

As shown in Figure 2A, the addition of SFLLR-NH_2_ (5 μM) to Ca^2+^- and Mg^2+^-free Hank's balanced saline supplemented with 10 mM HEPES pH 7.4 (CMF-HBSS+; bath solution) induced a biphasic DMR signal in the –tet cells. The positive (increasing) DMR (P-DMR) signal was followed by a sequential negative (decay) signal (N-DMR). The peak amplitude of the DMR signal in the –tet cells reached 141 ± 13.7 pm (N = 15). The DMR signal amplitude at the end of the measurement (36 min after the application of SFLLR-NH_2_) reached 103 ± 16.7 pm (N = 15). In the +tet cells, the application of SFLLR-NH_2_ induced an N-DMR signal followed by a weak P-DMR sequence (Figure 2A). The peak amplitude of the DMR signal in the +tet cells reached -83.3 ± 17.5 pm, and the amplitude at the end of the measurement was -70.8 ± 24.8 pm (N = 15). Both the peak amplitude and the amplitude of the DMR signal at the end of the measurement in the +tet cells were significantly (both P < 0.001) different from those in the –tet cells.

To our knowledge, DMR technology has not previously been used to monitor the effect of [Mg^2+^]_e_ (or other extracellular ions) on the integrated cellular response. Moreover, increased [Mg^2+^]_e_ and natural polyamines (spermine and spermidine) have been shown to activate IR without the presence of INS [30].

Therefore, we next tested whether the changing of [Mg^2+^]_e_ influenced the DMR profile of the –tet and +tet cells. During the measurements, the cells were bathed in CMF-HBSS+ plus Mg^2+^. The addition of Mg^2+^ (1, 3 or 10 mM) induced dose-dependent DMR signals in both the –tet and +tet cells. However, the amplitude and overall profile of the DMR signals acquired in the –tet cells were markedly different from those in the +tet cells. Whereas the DMR signals in the –tet cells were clearly positive (P-DMR) at all three [Mg^2+^]_e_, the DMR signals in the +tet cells started as P-DMR signals and quickly reverted to decaying N-DMR signal sequences at all three [Mg^2+^]_e_ (Figure 2B, Table 1).

**Table 1:** DMR signals presented as response in picometers (pm) and measured in –tet and +tet HEK293 cells at the maximum of P-DMR amplitude and at the time point of 36 min after application of the indicated [Mg^2+^]_e_, in the absence or presence of INS in the saline

INS (μU/mL)		0	400	0	400
Tet (1 μg/mL/15 hrs)		-	-	+	+
[Mg^2+^]_e_ (mM)	DMR (pm)				
1	P-DMR_max_	57.7 ± 4.38	59.8 ± 4.77	9.58 ± 4.72	19.9 ± 7.80
3	P-DMR_max_	104 ± 8.91	92.5 ± 7.97	25.9 ± 6.41	44.3 ± 7.94^*^
10	P-DMR_max_	256 ± 22.2	314 ± 26.7	109 ± 20.3	96.8 ± 26.8
1	36 min	57.7 ± 4.38	59.8 ± 4.77	-63.5 ± 5.85	-11.8 ± 10.2^***^
3	36 min	104 ± 8.91	92.5 ± 7.97	-52.8 ± 8.13	-23.4 ± 10.3^*^
10	36 min	256 ± 22.2	314 ± 26.7	-47.7 ± 6.77	-40.1 ± 15.3

N = 15 for each tested condition. Significance of pairwise comparison INS 0 μU/mL vs. INS 400 μU/mL in both –tet and +tet cells at all tested [Mg^2+^]_e_: P^*^ < 0.05, P^***^ < 0.001. Abbreviations: DMR, dynamic mass redistribution; INS, insulin; P-DMR_max_, maximal amplitude of positive DMR; tet, tetracycline.

We subsequently tested whether INS (400 μU/mL) quantitatively and/or qualitatively changed the DMR signals seen in the –tet and +tet cells upon the addition of 1, 3, or 10 mM Mg^2+^ to the bath solution. As demonstrated in Figure 2C and Table 1, the simultaneous application of INS and Mg^2+^ had no pronounced effect on the quantitative and/or qualitative features of the [Mg^2+^]_e_-dependent DMR signals in the –tet cells. However, in the +tet cells, the simultaneous application of INS and 3 mM Mg^2+^ led to a significant (P < 0.05) increase of the peak amplitude of the P-DMR signal (Figure 2C, Table 1). The application of INS together with 1 or 3 mM Mg^2+^ led to a significant (P < 0.001 and P < 0.05, respectively) reduction of the amplitude of the N-DMR signal at the end of the measurement (time point 36 min; Figure 2C, Table 1). The simultaneous application of INS and 10 mM Mg^2+^ had no significant effect on the amplitude of the [Mg^2+^]_e_-dependent DMR signal at the time point 36 min in +tet cells (Figure 2C, Table 1).

**Figure 3:**
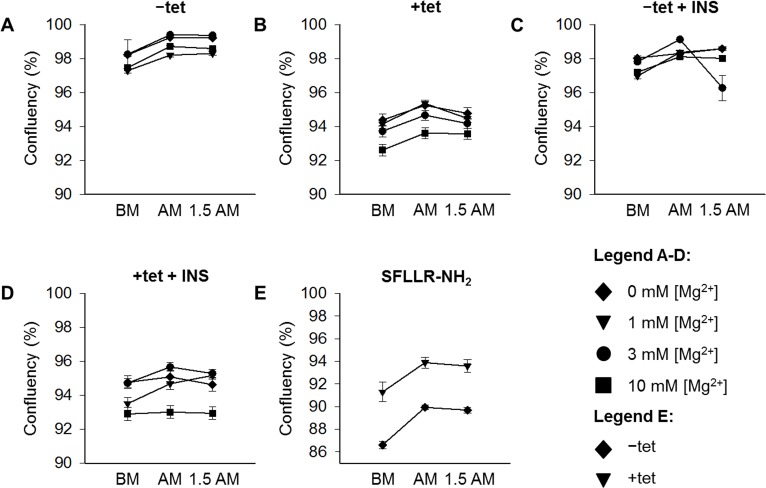
Cell confluence assessed by using in-well imaging before the application of the particular effector(s), immediately after measurements, and 1. 5 hour after the end of measurements Measurements were performed with –tet **(A, C, E)** and with +tet HEK293 cells **(B, D, E)** without INS (A, B) or with INS (400 μU/mL; C, D) in CMF-HBSS+. [Mg^2+^]_e_ are indicated. Data are presented as means (for A – D, N = 12 – 15; for E, N = 9) ± SE. Abbreviations: 1.5 AM, 1.5 hrs after measurement; AM, after measurement; BM, before measurement; INS, insulin; SFLLR-NH_2_, protease-activated receptor 1 activating peptide; tet, tetracycline.

All DMR measurements were accompanied by a cell confluence assessment by using in-well imaging before the application of the particular effector, immediately after measurements and at 1.5 hours after the end of measurements (Supplementary Figure 1). Figure 3 demonstrates that none of the interventions and measurements had any significant effect on cell confluence and viability.

Taken together, our data reveal that the overexpression of A1 changes conspicuously the SFLLR-NH_2_- or [Mg^2+^]_e_-specific DMR fingerprint of HEK293 cells, indicating a complex physiological difference between the –tet and +tet cells. Furthermore, INS has an effect on the [Mg^2+^]_e_-dependent DMR signal in the +tet cells at [Mg^2+^]_e_ of 1 and 3 mM.

### Overexpression of A1 and variation of [Mg^2+^]_e_ modulate Akt/PKB signaling

**Figure 4:**
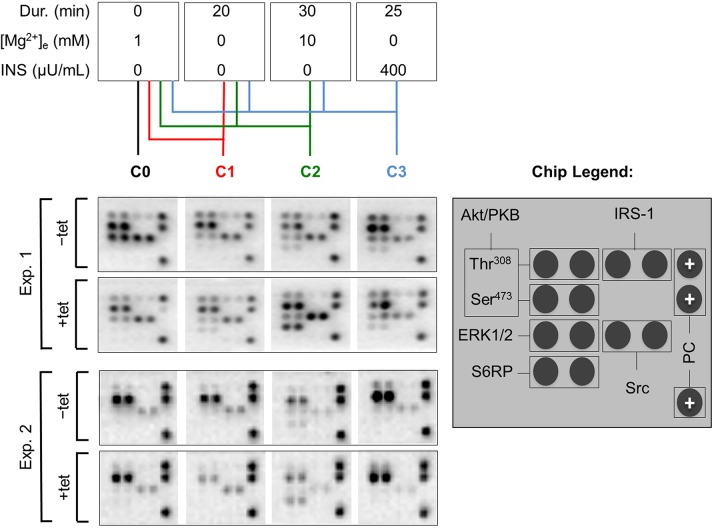
PathScan^®^ RTK Signaling Antibody Arrays (PRSA) displaying phosphorylation of respective phospho-substrates in –tet (control) and +tet (SLC41A1-overexpressing) HEK293 cells treated with C0, C1, C2, or C3 Two examples out of six independent experimental series are shown. Abbreviations: Akt/PKB, protein kinase B; C, condition; Src, proto-oncogene tyrosine-protein kinase Src; Dur., duration; Erk1/2, extracellular signal-regulated kinase 1/2; INS, insulin; IRS-1, insulin receptor substrate 1; PC, positive control; S6RP, S6 ribosomal protein; tet, tetracycline.

**Figure 5:**
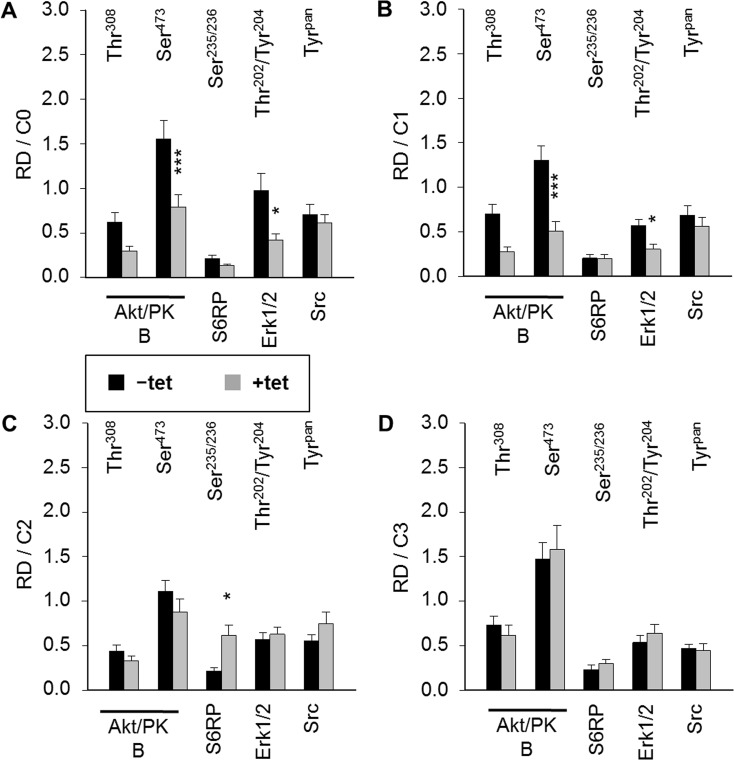
Pairwise comparison of averaged relative densities of particular phospho-signals (Akt/PKB-Thr^308^, Akt/PKB-Ser^473^, S6RP-Ser^235/236^, Erk1/2-Thr^202^/Tyr^204^, Src-Tyr^pan^) in –tet (control) and +tet (SLC41A1-overexpressing) HEK293 cells treated with **(A)** C0, **(B)** C1, **(C)** C2, or **(D)** C3, as obtained with PathScan^®^ RTK Signaling Antibody Array (see Figure 4 for details). Each averaged density value results from six independent biological experiments (N/n = 6/12; respective phospho-signals were detected on chips in duplicate). Data are presented as means ± SE; ^*^P < 0.05, ^***^P < 0.001. Abbreviations: Akt/PKB, protein kinase B; C, condition; Erk1/2, extracellular signal-regulated kinase 1/2; RD, relative density; S6RP, S6 ribosomal protein; Src, proto-oncogene tyrosine-protein kinase Src; tet, tetracycline.

As we had observed that both A1 overexpression and [Mg^2+^]_e_ induce and/or modify the DMR signal, we next examined whether the overexpression of A1 and/or variation of [Mg^2+^]_e_ influenced any of the 11 important signaling nodes included in the PathScan^®^ RTK Signaling Antibody Array (PRSA). As shown in Figures 4 and 5A, prominent signals corresponding to phosphorylated Akt/PKB-Thr^308^, Akt/PKB-Ser^473^, S6RP-Ser^235/236^, Erk1/2-Thr^202^/Tyr^204^, and Src-Tyr^pan^ were detected in the –tet and +tet cells, indicating that these kinases and their adjacent signaling cascades play an important role in the basal processes in these cells. Furthermore, the overexpression of A1 (15 hrs, condition 0 (C0)) significantly attenuated (by 49.1± 9%; P < 0.001) the phosphorylation of Akt/PKB-Ser^473^ in +tet cells when compared with –tet cells (Figures 4 and 5A). Moreover, the phosphorylation of Erk1/2-Thr^202^/Tyr^204^ was significantly attenuated (by 56.8 ± 7%; P < 0.05) in +tet cells when compared with –tet cells (Figures 4 and 5A). No statistical differences were detected between the levels of phosphorylation of Akt/PKB-Thr^308^, S6RP-Ser^235/236^, and Src-Tyr^pan^ in –tet and +tet cells (Figure 5A).

We also examined whether the variation of [Mg^2+^]_e_ differently influenced the phosphorylation of Akt/PKB-Thr^308^, Akt/PKB-Ser^473^, S6RP-Ser^235/236^, Erk1/2-Thr^202^/Tyr^204^, Src-Tyr^pan^, or any of the other important signaling nodes included in PRSA in –tet and +tet cells (Figure 5). First, we incubated –tet and +tet cells in Mg^2+^-free salt solution (CMF-HBSS+) for 20 min. This treatment (condition 1 (C1)) led to a significant (P < 0.001 and P < 0.05, respectively) reduction (by 61 ± 8% and 47 ± 9%, respectively) of Akt/PKB-Ser^473^ and Erk1/2-Thr^202^/Tyr^204^ phosphorylation in +tet cells when compared with –tet cells (Figure 5B). No significant differences in the levels of phosphorylation of Akt/PKB-Thr^308^, S6RP-Ser^235/236^, and Src-Tyr^pan^ were detected between –tet and +tet cells (Figure 5B). Next, after C1, we provided –tet and +tet cells with HBSS+ supplemented with 10 mM Mg^2+^ for 30 min (condition 2 (C2)). This led to a significantly higher (by 186 ± 56%; P < 0.05) phosphorylation of S6RP-Ser^235/236^ in the +tet cells than in the –tet cells (Figure 5C). The levels of phosphorylation of Akt/PKB-Thr^308^, Akt/PKB-Ser^473^, Erk1/2-Thr^202^/Tyr^204^, and Src-Tyr^pan^ were not significantly different between –tet and +tet cells after C2 (Figure 5C). As we had demonstrated that INS influenced the performance of A1 via classic INS signaling involving IRTK – PI3K – Akt/PKB [11], we provided –tet and +tet cells (after C1 + C2) with CMF-HBSS+ containing INS (400 μU/mL) for 25 min (condition 3 (C3)). This treatment led to comparable levels of phosphorylation of all detectable phospho-targets in +tet and –tet cells (Figure 5D).

These data clearly indicate that A1 overexpression influences the phosphorylation (and, thus, the activity) of Akt/PKB-Ser^473^ and Erk1/2-Thr^202^/Tyr^204^ in Mg^2+^-free medium and in medium containing the physiological [Mg^2+^] (1 mM). Furthermore, they show that the presence of high 10 mM [Mg^2+^]_e_ enhances the phosphorylation of S6RP-Ser^235/236^ in the +tet cells. Application of INS (400 μU/mL; 25 min) erases any differences in the phosphorylation status of Akt/PKB-Ser^473^ and Erk1/2-Thr^202^/Tyr^204^ resulting from the different expression niveaus of A1 in –tet and +tet cells, as seen in Mg^2+^-free medium and medium supplied with 1 mM Mg^2+^.

**Figure 6:**
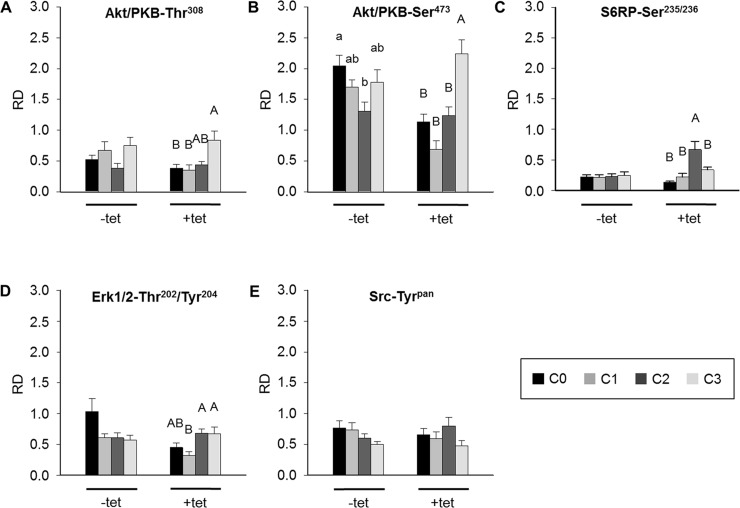
Between-treatments (C0, C1, C2, C3; see Figure 4 for details) comparison of averaged relative densities of **(A)** Akt/PKB-Thr^308^ phospho-signals, **(B)** Akt/PKB-Ser^473^ phospho-signals, **(C)** S6RP-Ser^235/236^ phospho-signals, **(D)** Erk1/2-Thr^202^/Tyr^204^ phospho-signals, and **(E)** Src-Tyr^pan^ phospho-signals in –tet (control) and +tet (SLC41A1-overexpressing) HEK293 cells, as obtained with PathScan^®^ RTK Signaling Antibody Array (PRSA). Each averaged density value results from six independent biological experiments (N/n = 6/12 (respective phospho-signals were detected on chips in duplicate)). Data are presented as means ± SE; a vs. b P < 0.05. Abbreviations: Akt/PKB, protein kinase B; C, condition; Erk1/2, extracellular signal-regulated kinase 1/2; RD, relative density; S6RP, S6 ribosomal protein; Src, proto-oncogene tyrosine-protein kinase Src; tet, tetracycline.

The sequential experimental design allowed the pairwise comparisons of the effects of particular treatments (C0, C1, C2, C3) on the phosphorylation of the respective kinases in –tet and +tet cells. None of the treatments had a significant effect on the levels of phosphorylation of Akt/PKB-Thr^308^ in –tet cells (Figure 6A). A significant (P < 0.05) increase of Akt/PKB-Thr^308^ phosphorylation was evoked by C3 when compared with C0 and C1, but not with C2, in +tet cells (Figure 6A). A significantly (P < 0.05) lower level of Akt/PKB-Ser^473^ phosphorylation was detected in –tet cells after C2 when compared with that after C0 (Figure 6B). In +tet cells, a significantly (P < 0.001) higher niveau of Akt/PKB-Ser^473^ phosphorylation was detected after C3 when compared with C0, C1, and C2 (Figure 6B). No significant changes of S6RP-Ser^235/236^ phosphorylation were detected upon any of the applied treatments in the –tet cells (Figure 6C). Treatment C2 led to a significantly (P < 0.001) increased phosphorylation of S6RP-Ser^235/236^ when compared with treatments C0, C1, and C3 in +tet cells (Figure 6C). Moreover, no significant changes of Erk1/2-Thr^202^/Tyr^204^ phosphorylation were detected following any of the applied treatments in –tet cells (Figure 6D). Erk1/2-Thr^202^/Tyr^204^ phosphorylation was significantly (P < 0.05) reduced after C1 when compared with that after C2 or C3 in +tet cells (Figure 6D). None of the treatments induced a significant change of Src-Tyr^pan^ phosphorylation in –tet or +tet cells (Figure 6E).

Taken together, the above data indicate that the modulation of [Mg^2+^]_e_ and/or exposure to a high concentration of INS significantly affect the levels of Akt/PKB-Thr^308^ phosphorylation in +tet cells, of the Akt/PKB-Ser^473^ phosphorylation in both –tet and +tet cells, of the S6RP-Ser^235/236^ phosphorylation in +tet cells, and of the Erk1/2-Thr^202^/Tyr^204^ phosphorylation in +tet cells.

As we observed the most prominent effects of A1 overexpression and/or modulation of [Mg^2+^]_e_ on the phosphorylation of Akt/PKB and Erk1/2, we decided to verify the PRSA results with Western blot (WB) analysis and densitometry.

**Figure 7:**
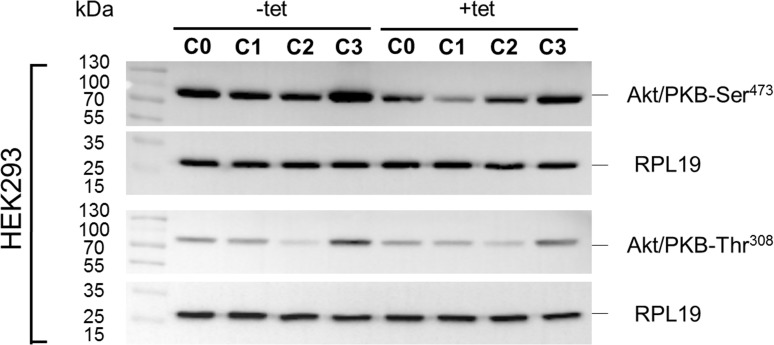
WB analysis of the phosphorylation status of Akt/PKB-Thr^308^ and Akt/PKB- Ser^473^ in response to the treatment conditions C0, C1, C2, or C3 (see Figure 4 for details) in –tet (control) and +tet (SLC41A1-overexpressing) HEK293 cells One representative experiment of, in total, five independent biological experiments is shown. RPL19 was used as a loading reference and was detected subsequent to the phospho-signals on the same blots. Abbreviations: Akt/PKB, protein kinase B; C, condition; RPL19, 60S ribosomal protein L19; tet, tetracycline; WB, Western blot.

**Figure 8:**
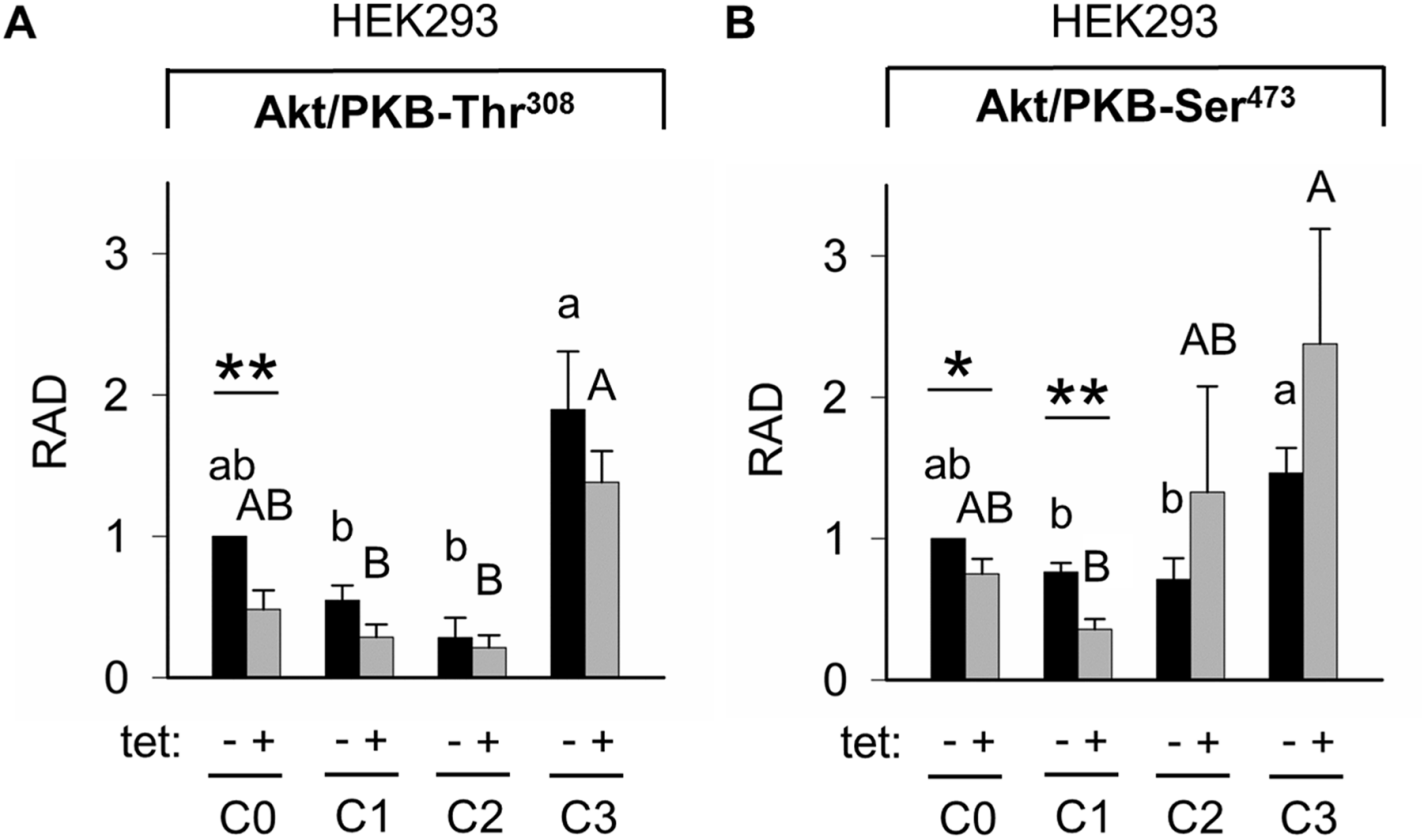
Pairwise comparison of relative adjusted densities of Akt/PKB-Thr^308^ **(A)** and Akt/PKB-Ser^473^ **(B)** phospho-signals and between-treatments (C0, C1, C2, C3; see Figure 4 for details) comparison of relative adjusted densities of Akt/PKB-Thr^308^ (A) and Akt/PKB-Ser^473^ (B) phospho-signals in –tet (control) and +tet (SLC41A1-overexpressing) HEK293 cells, obtained with WB. Data are presented as means (N = 5) ± SE. Pairwise comparisons: Significance is indicated (^*^P < 0.05, ^**^P < 0.01). Between-treatment comparisons: Labeled means without a common letter differ (P < 0.05). Small letters were used for the between-treatment comparisons in –tet group and capital letters in +tet group. Abbreviations: Akt/PKB, protein kinase B; C, condition; RAD, relative adjusted density; tet, tetracycline; WB, Western blot.

Whereas PRSA had suggested only a numerical reduction in Akt/PKB-Thr^308^ phosphorylation by A1 overexpression, the WB analysis revealed that Akt/PKB-Thr^308^ was significantly (P < 0.01) less phosphorylated in cells overexpressing A1 and grown in the presence of [Mg^2+^]_e_ at 1 mM (C0) when compared with –tet cells under the same conditions (Figures 7 and 8A). Coherent with the results obtained with PRSA, the WB data show that the phosphorylation of Akt/PKB-Ser^473^ is indeed significantly (P < 0.05 and P < 0.01) attenuated in +tet cells when compared with that in –tet cells, under both C0 and C1 (Figures 7 and 8B).

The comparisons of Akt/PKB-Thr^308^ phosphorylation between treatments revealed the significantly (P < 0.05) higher level of Thr^308^ phosphorylation in –tet cells after C3 when compared with C1 and C2 (Figure 8A). This was also the case in +tet cells (Figure 8A). In the case of Akt/PKB-Ser^473^, the comparison of its phosphorylation between treatments showed a significantly (P < 0.05) increased niveau of phospho-Ser^473^ in –tet cells after C3 when compared with C1 and C2 (Figure 8B). In +tet cells after C3, the level of Ser^473^ phosphorylation was significantly (P < 0.05) higher than that after C1 (Figure 8B).

**Figure 9:**
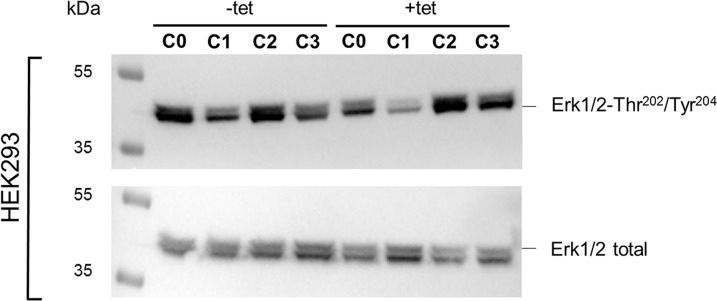
WB analysis of the phosphorylation status of Erk1/2-Thr^202^/Tyr^204^ in response to the treatment conditions C0, C1, C2, or C3 (see Figure 4 for details) in –tet (control) and +tet (SLC41A1-overexpressing) HEK293 cells One representative experiment of, in total, five independent biological experiments is shown, in which total Erk1/2 and the phospho-signals of Erk1/2-Thr^202^/Tyr^204^ were detected in parallel. Abbreviations: C, condition; Erk1/2, extracellular signal-regulated kinase 1/2; tet, tetracycline; WB, Western blot.

**Figure 10:**
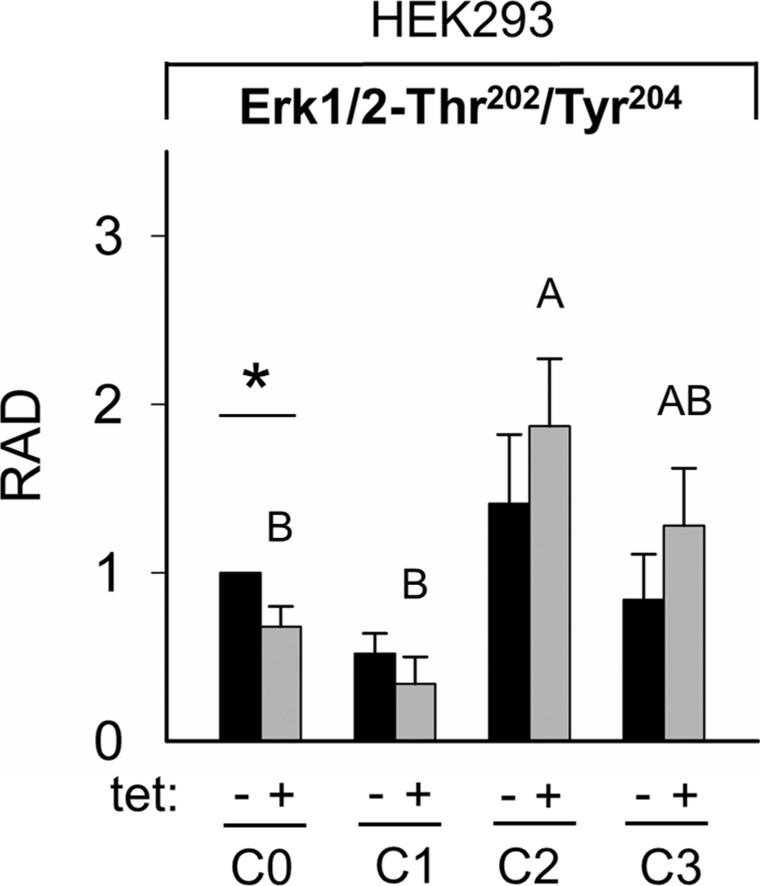
Pairwise comparison of relative adjusted densities of Erk1/2-Thr^202^/Tyr^204^ phospho-signals and between-treatments (C0, C1, C2, C3; see Figure 4 for details) comparison of relative adjusted densities of Erk1/2-Thr^202^/Tyr^204^ phospho-signals in –tet (control) and +tet (SLC41A1-overexpressing) HEK293 cells, obtained with WB Data are presented as means (N = 5) ± SE. Pairwise comparisons: significance is indicated (^*^P < 0.05). Between-treatment comparisons: Labeled means without a common letter differ (P < 0.05). Small letters were used for the between-treatment comparisons in –tet group and capital letters in +tet group. Abbreviations: C, condition; Erk1/2, extracellular signal-regulated kinase 1/2; RAD, relative adjusted density; tet, tetracycline; WB, Western blot.

The WB analysis confirmed the PRSA results showing that Erk1/2-Thr^202^/Tyr^204^ phosphorylation was significantly (P < 0.05) reduced in cells overexpressing A1 and grown in the presence of [Mg^2+^]_e_ at 1 mM (C0) when compared with –tet cells under the same conditions (Figures 9 and 10). Although the PRSA data showed a significant reduction of Erk1/2-Thr^202^/Tyr^204^ phosphorylation in +tet cells under C1 (compared with –tet cells under C1), the reduction of Erk1/2-Thr^202^/Tyr^204^ phosphorylation in +tet cells under C1 as detected with WB analysis was not significant (P = 0.17) when compared with –tet cells under C1.

The comparisons of the Erk1/2-Thr^202^/Tyr^204^ phosphorylation between treatments revealed the significantly (P < 0.05) higher level of Thr^202^/Tyr^204^ phosphorylation in +tet cells after C2 when compared with C0 and C1 (Figure 10). However, this was not the case in –tet cells (Figure 10).

In summary, the densitometric WB and PRSA data demonstrated similar changes in the phosphorylation profile (and thus activity) of Akt/PKB and Erk1/2 in response to the expression status of A1.

Next, we examined whether the above-described attenuation of the phosphorylation of Akt/PKB triggered by A1 overexpression is a common phenomenon in various cell types, or whether it is limited to HEK293 cells only. SH-SY5Y neuroblastoma-derived cells were transfected with empty vector (SH-SY5Y-A^-^ cells) or vector carrying and constitutively expressing A1 (SH-SY5Y-A^+^ cells).

Coherent with data acquired from HEK293 cells, the WB analysis revealed that Akt/PKB-Thr^308^ was significantly (P < 0.01) less phosphorylated in SH-SY5Y-A^+^ cells overexpressing A1 and grown in the presence of [Mg^2+^]_e_ at 1 mM (C0) when compared with SH-SY5Y-A^-^ cells under the same conditions (Supplementary Figures 2 and 3A). Moreover, the phosphorylation of Akt/PKB-Ser^473^ was significantly (P < 0.01 and P < 0.05) attenuated in SH-SY5Y-A^+^ cells when compared with that in SH-SY5Y-A^-^ cells, under both C0 and C1 (Supplementary Figures 2 and 3B). No significant differences in phosphorylation of both Akt/PKB-Thr^308^ and Akt/PKB-Ser^473^ were detected between SH-SY5Y-A^-^ and SH-SY5Y-A^+^ cells under either C2 or C3 (Supplementary Figures 2, 3A and 3B).

Between-treatments comparisons of Akt/PKB-Thr^308^ phosphorylation revealed the significantly (P < 0.05) higher level of Thr^308^ phosphorylation in SH-SY5Y-A^-^ cells after C3 when compared with C1 and C2 and under C0 when compared with C2 (Supplementary Figure 3A). In SH-SY5Y-A^+^ cells, the latter was observed, except that no significant difference was detected between C0 and C2 (Supplementary Figure 3A). A comparison of Akt/PKB-Ser^473^ phosphorylation between treatments showed significantly (P < 0.05) increased levels of phosphorylated Ser^473^ in both SH-SY5Y-A^-^ and SH-SY5Y-A^+^ cells after C3 when compared with C1 and C2, and in C0 when compared with C2 (Supplementary Figure 3B).

The Akt/PKB-Thr^308^ and -Ser^473^ phosphorylation profiles in HEK293 cells and in SH-SY5Y cells in response to the expression status of SLC41A1 and the status of the extracellular and intracellular Mg^2+^ largely overlaps. The levels of phosphorylation of both Thr^308^ and Ser^473^ are much higher under C3 in SH-SY5Y cells when compared with HEK293 cells suggesting (Figure 8A and 8B vers. Supplementary Figure 3A and 3B) that SH-SY5Y cells are obviously better responders regarding the INS stimulation.

Consequently, we examined the phosphorylation status of Erk1/2-Thr^202^/Tyr^204^ in SH-SY5Y-A^-^ and SH-SY5Y-A^+^ cells. Similar to HEK293 cells, we detected a significant (P < 0.001) reduction of Erk1/2-Thr^202^/Tyr^204^ phosphorylation related to the overexpression of A1 in SH-SY5Y-A^+^ cells when compared with SH-SY5Y-A^-^ under C0 (Supplementary Figures 4 and 5). A significantly (P < 0.05) higher phosphorylation of Erk1/2-Thr^202^/Tyr^204^ was detected in SH-SY5Y-A^+^ under C3 when compared with SH-SY5Y-A^-^ cells treated in the same way (Supplementary Figure 5). This indicates that the overexpression of A1 facilitates the effect of INS on Erk1/2 phosphorylation and, thus, its activity in SH-SY5Y cells.

Statistical analysis for the between-treatment comparisons were missing in Supplementary Figure 5 and are now included in the new Supplementary Figure 5 which is attached. However, the same comparison in SH-SY5Y-A^+^ cells revealed significantly (P < 0.05) higher Erk1/2-Thr^202^/Tyr^204^ phosphorylation in cells treated under C3 compared with cells treated under C0 or C1 (Supplementary Figure 5).

Finally, we examined the correlation between A1 overexpression and the phosphorylation status of Akt/PKB and ERK1/2 in HeLa adenocarcinoma-derived cells with tet inducible expression of A1. Under our experimental conditions, basal phosphorylation of Akt/PKB at Ser^473^ and, in particular, at Thr^308^ was extremely low in this cell line. Low basal Akt/PKB phosphorylation in HeLa cells was also found by other authors by using the phospho-specific antibodies used in this study [31]. We therefore compared Akt/PKB phosphorylation between –tet and +tet cells in this cell line only under C3, as only INS treatment stimulated the sufficient detectable phosphorylation of both Thr^308^ and Ser^473^. As shown in Supplementary Figures 6 and 7, the INS-induced phosphorylation of Thr^308^ was significantly (P < 0.05) lower in +tet cells in comparison with in –tet cells. Moreover, phosphorylation of Ser^473^ was significantly (P < 0.01) lower in +tet cell than in –tet cells (Supplementary Figures 6 and 7).

The phosphorylation of Erk1/2-Thr^202^/Tyr^204^ in HeLa cells was well detectable under all tested conditions. Erk1/2 was significantly (P < 0.05) less phosphorylated in +tet cells when compared with –tet cells (Supplementary Figures 8 and 9). The comparison of Erk1/2 phosphorylation between –tet and +tet cells under C1, C2, and C3 revealed no significant differences (Supplementary Figures 8 and 9).

In between-treatments comparisons of Erk1/2-Thr^202^/Tyr^204^ phosphorylation in –tet cells, we found a significant (P < 0.05) difference between treatment C0 and C1 (Supplementary Figure 9). The same comparison in +tet cells revealed no significant differences between particular conditions (Supplementary Figure 9).

In brief summary, WB analyses revealed that, in HEK293 and SH-SY5Y cells, the overexpression of A1 correlated with the decrease of Akt/PKB and Erk1/2-Thr^202^/Tyr^204^ at a physiological [Mg^2+^]_e_ of 1 mM (C0). The Akt/PKB phosphorylation was below the detection limit of WB in HeLa cells under all tested [Mg^2+^]_e_ (C0, C1, and C2). However, it became detectable upon use of hyperinsulinemic [INS]_e_ of 400 μU/mL with no Mg^2+^ being present in the bath solution, and overexpression of A1 under this condition lead to a significantly (P < 0.05, P < 0.01) lower Akt/PKB-Thr^308^ and -Ser^473^ phosphorylation when compared with Akt/PKB-Thr^308^ and -Ser^473^ phosphorylation in –tet HeLa cells.

### Overexpression of SLC41A1 clearly modifies [Mg^2+^]_i_ at all tested conditions

In our previous work, we have demonstrated that SLC41A1 is a functional NME that operates primarily in efflux mode under normal physiological conditions [2, 3, 4, 11]. However, A1 may operate also in reverse mode, thus conducting Mg^2+^ influx, but only if a sufficiently large [Mg^2+^]_e_ is provided [5, 6, 13]. Here, we measured [Mg^2+^]_i_ in –tet and +tet HEK293 cells after treatment C0, C1, C2, and C3. The results are summarized in Table 2. Overexpression of A1 (15 hrs) lead to a significant (P < 0.001) reduction of [Mg^2+^]_i_ when compared with [Mg^2+^]_i_ in control –tet cells (both –tet and +tet cells were measured at [Mg^2+^]_e_ = 1 mM; C0). +Tet cells incubated 30 min in CMF-HBSS+ ([Mg^2+^]_e_ = 0 mM; C1) had significantly (P < 0.001) lower [Mg^2+^]_i_ when compared with [Mg^2+^]_i_ in control –tet cells treated in the same way. The [Mg^2+^]_i_ determined in +tet cells treated under C2 (cells were bathed for 30 min in CMF-HBSS+ and 30 min in HBSS+ containing [Mg^2+^]_e_ = 10 mM) were significantly (P < 0.01) higher than the [Mg^2+^]_i_ in –tet cells treated under C2. Finally, we measured [Mg^2+^]_i_ in –tet and +tet cells that were bathed for 30 min in CMF-HBSS+ followed by a 30 min bath in HBSS+ containing [Mg^2+^]_e_ = 10 mM and a 25 minutes bath in CMF-HBSS+ supplemented with 400 μU/mL of INS (C3). As is evident from Table 2, the [Mg^2+^]_i_ measurements in –tet cells were not significantly different from those measured in +tet cells.

**Table 2:** [Mg^2+^]_I_ measured in –tet and +tet HEK293 cells under treatments C0 through C3

Condition	-tet		+tet		-tet vs. +tet
	N	[Mg^2+^]_i_ (mM)	N	[Mg^2+^]_i_ (mM)	
C0	17	0.31 ± 0.08	15	0.23 ± 0.03	P < 0.001
C1	18	0.21 ± 0.07	18	0.05 ± 0.02	P < 0.001
C2	10	0.57 ± 0.06	10	0.77 ± 0.04	P < 0.01
C3	10	0.75 ± 0.07	10	0.67 ± 0.04	P = 0.39

Conditions: C0, [Mg^2+^]_i_ was measured in –tet and +tet cells immediately after 30-min loading with mag-fura 2 fluorescent probe upon presence of [Mg^2+^]_e_ = 1 mM; C1, [Mg^2+^]_i_ was measured in –tet and +tet cells after 30-min loading with mag-fura 2 fluorescent probe in CMF-HBSS+; C2, [Mg^2+^]_i_ was measured in –tet and +tet cells after 30-min loading with mag-fura 2 fluorescent probe in CMF-HBSS+ followed by 30-min incubation of the cells in HBSS+ containing 10 mM Mg^2+^; C3, [Mg^2+^]_i_ was measured in –tet and +tet cells after 30-min loading with mag-fura 2 fluorescent probe in Mg^2+^-free HBSS+ followed by 30-min incubation of the cells in HBSS+ containing 10 mM Mg^2+^ and successively by 25-min incubation of the cells in CMF-HBSS+ provided with INS (400 μU/mL). Abbreviations: C, condition; CMF-HBSS+, Ca^2+^- and Mg^2+^-free Hank's balanced salt solution plus 10 mM HEPES pH 7.4; tet, tetracycline.

From our previous, the expression levels of SLC41A1 significantly influence the status of [Mg^2+^]_i_ under C0, C1, and C2. In contrast, under C3 (hyperinsulinemic concentration of INS), the SLC41A1 expression level did not significantly influence [Mg^2+^]_i_.

The multiple pairwise comparison of [Mg^2+^]_i_ between particular conditions within the group of –tet cells (summarized in Table 3) revealed no significant difference between the [Mg^2+^]_i_ of cells under C1 when compared with [Mg^2+^]_i_ of cells treated under C0. However, [Mg^2+^]_i_ of cells under C1 were significantly (P < 0.05) lower when compared with [Mg^2+^]_i_ of cells under C2 or C3. The concentrations of intracellular Mg^2+^ measured in cells under C2 were not significantly different from Mg^2+^ concentrations determined in cells under C0 or C3; [Mg^2+^]_i_ of cells under C0 were significantly (P < 0.05) lower when compared with [Mg^2+^]_i_ of cells under C3.

**Table 3:** Pairwise comparisons of [Mg^2+^]_i_ measured separately in –tet HEK293 cells or in +tet HEK293 cells under C0 through C3

tet	-tet				+tet			
Condition	C0	C1	C2	C3	C0	C1	C2	C3
[Mg^2+^]_i_(mM)	0.31 ± 0.08	0.21 ± 0.07	0.57 ± 0.06	0.75 ± 0.07	0.23 ± 0.03	0.05 ± 0.02	0.77 ± 0.04	0.67 ± 0.04
Significance (defined by P < 0.05)	bc	c	ab	a	B	C	A	AB

Conditions were as previously described in legend for Table 2. Letter code indicates statistically significant differences between compared datasets. Abbreviations: C, condition; tet, tetracycline.

The multiple pairwise comparison of [Mg^2+^]_i_ between particular conditions within the group of +tet cells (summarized in Table 3) showed that [Mg^2+^]_i_ of cells under C1 were significantly (P < 0.05) lower than [Mg^2+^]_i_ of cells treated under C0, C2, or C3. [Mg^2+^]_i_ of cells under C2 were significantly (P < 0.05) higher when compared with [Mg^2+^]_i_ of cells under C0 and C1, but not C3. [Mg^2+^]_i_ of cells under C3 were significantly (P < 0.05) higher than [Mg^2+^]_i_ of cells under C1, but not C0 or C2.

## DISCUSSION

The involvement of Mg^2+^ in more than 300 distinctive enzymatic reactions and key cellular processes substantiates the need for the efficient cellular regulation of [Mg^2+^]_i_ [1]. Regulated active Mg^2+^ transport mechanisms are essential for the maintenance of IMH [7].

Extracellular signals (e.g., hormones) and intracellular signaling play an important role in the regulation of Mg^2+^ transporters and, thus, IMH [1]. However, the paucity of information concerning the molecular biology of the respective cellular Mg^2+^ transport systems makes our understanding of the role of cellular signaling in IMH incomplete.

NME A1 is a key component of IMH regulation and is still the only known ubiquitously expressed Mg^2+^ efflux system in cells [4–7, 10, 11, 13]. Recently, we have demonstrated that INS regulates the NME activity of A1 via the “classic” signaling cascade IRTK – PI3K – Akt/PKB [13]. Moreover, ANG has been implicated in the regulation of NME [32]. Both INS and ANG influence intracellular cAMP levels and, consequently, the PKA activity that triggers A1 activity [13, 33].

Overexpression of A1 results in a decrease of the basal [Mg^2+^]_i_ in cells cultured at the physiological [Mg^2+^]_e_ of 1 mM (Tables 2 and 3) [4, 5]. Goytain and Quamme have demonstrated that a restriction of Mg^2+^ intake induces the upregulation of A1 expression in mice [34]. Nothing is known about the signaling involved in the regulation of *A1* transcription, apart from the work of Romanuik and Hwang [24] who have identified *A1* as being an androgen-responsive gene and who have therefore assumed the existence of *ARE*s in regulatory sequences surrounding the coding regions of *A1* [24, 25]. Furthermore, nothing is known about the impact of A1 overexpression on cellular signaling and complex cell physiology.

Our data (Figure 2) demonstrate that the overexpression of A1 completely changes the SFLLR-NH_2_- and [Mg^2+^]_e_-induced DMR fingerprint of HEK293 cells, thus identifying the A1 expression level as being a *bona fide* discriminant able to modify complex cellular responses to external signals translated into a measurable DMR signal. Indeed, these data indicate that not only extracellular signals and adjacent afferent signaling influence *A1* (or A1) at the transcriptional and the functional levels, but also *vice versa*, i.e., that levels of *A1* transcription influence intracellular signaling.

Akt/PKB has a central role in cellular signaling and in the regulation of proliferation, growth, and apoptosis [35, 36]. The phosphorylation of Thr^308^ activates Akt/PKB, although the phosphorylation of Thr^308^ and Ser^473^ is required for its full activity. Significantly, the phosphorylation of Ser^473^ alone has little effect on Akt/PKB activity [35, 36]. Several studies have implicated Mg^2+^ in the regulation of Akt/PKB activity [37, 38]. Krueger et al. have demonstrated that elevated [Mg^2+^]_e_ (3.3 mM) increases cell proliferation in neural cells in culture and have observed that increased Akt/PKB activation, comparable with activation by INS (200 nM), follows Mg^2+^ treatment [37]. The activity of Akt/PKB results from the orchestrated actions of kinase PDK-1, which phosphorylates Thr^308^; kinase complex mTOR/RICTOR/GβL, which phosphorylates Ser^473^; kinase PI3K, which phosphorylates both Thr^308^ and Ser^473^ in Akt/PKB isoform 1 or Ser^474^ alone in isoform 2; phosphatase PP2A, which dephosphorylates Thr^308^; and the PHLPP phosphatases, which dephosphorylate Ser^473^ [39–44]. PHLPP is a member of the protein phosphatase Mg^2+^-activated (PPM) subfamily of phosphatases. It requires Mg^2+^ for its catalytic activity [44]. Mg^2+^ also plays a role in the activation of PP2A, but only in complex with ATP [45]. Low [Mg^2+^]_e_ is also assumed to activate PI3K [46].

Here, we have demonstrated that the overexpression of NME A1 significantly attenuates the phosphorylation of Akt/PKB at both Thr^308^ and Ser^473^ (Figures 5A, 5B, 8A, 8B, Supplementary Figure 3). These data nicely complement the aforementioned work of Krueger and colleagues [37]. However, the above also indicates that the low [Mg^2+^]_i_ that results from the overexpression of A1 should down-regulate PHLPP, and perhaps PP2A, and activate PI3K to increase the phosphorylation of both Thr^308^ and Ser^473^ or at least to keep their phosphorylation status at a sustained level (Supplementary Figure 10A). Hence, an as yet unknown regulatory mechanism must exist that is potent enough to increase the activity of PHLPP and PP2A and to decrease that of PI3K, PDK-1, and mTOR/RICTOR/GβL at the lower [Mg^2+^]_i_ induced by the overexpression of A1 (Supplementary Figure 10B). Whether such a regulator is the recently identified Mg^2+^ homeostatic factor CNNM2 remains to be examined [47].

Further, our data indicate that the variation of extracellular Mg^2+^ (C1, C2) also contributes to the modulation of the Akt/PKB phosphorylation on Thr^308^ and Ser^473^ in both the cells with endogenous A1 expression and A1-overexpressing cells, although the expression status of A1 remains a superior discriminant compared with the status of [Mg^2+^]_e_. Our results also suggest that INS stimulates the phosphorylation of both Thr^308^ and Ser^473^ independently of the presence of Mg^2+^ in the extracellular solution. The latter is also supported by the observation that, irrespective of A1 expression status, the presence of [Mg^2+^]_e_ = 10 mM (C2) was insufficient to increase the phosphorylation of Akt/PKB (at both Thr^308^ and Ser^473^) beyond the WB detection threshold in HeLa cells, whereas the hyperinsulinemic concentration of INS induced a detectable massive phosphorylation of both Thr^308^ and Ser^473^ of Akt/PKB in these cells, even without presence of Mg^2+^ in external solution (Supplementary Figures 6 and 7).

Notably, Erk1/2-Thr^202^/Tyr^204^ phosphorylation is significantly reduced in +tet HEK293 and in +tet HeLa and SH-SY5Y-A^+^ cells in the presence of physiological Mg^2+^ (1 mM) or with no Mg^2+^ (only in HEK293 cells) in the bath solution (Figures 5A, 5B, 10, Supplementary Figures 5 and 9). The phosphorylation of both Thr^202^ and Tyr^204^ residues is required for its activity [48]. Therefore, we can conclude that the overexpression of A1 contributes to the reduction of Erk1/2 kinase activity and thus to the modulation of its signaling in processes such as proliferation and/or apoptotic events in cells [49, 50]. The Ras – Erk1/2 and PI3K – Akt/PKB – mTORC1 pathways regulate each other via cross-inhibition and cross-activation [51]. Our data indicate that the lack of [Mg^2+^]_i,_ caused by the overexpression of A1 in the +tet cells bathed in medium containing no Mg^2+^ or 1 mM Mg^2+^ leads to an attenuation of both cascades (Tables 2 and 3) [4, 5]. Indeed, one can speculate that oscillations of [Mg^2+^]_i_ play a critical role in the regulation of both the aforementioned cascades and their crosstalk. Hence, from a larger perspective, A1 can be assumed to play a significant role in the regulation of Erk1/2 and Akt/PKB signaling and their orchestration in basic cellular processes, such as proliferation and apoptosis.

Ser/Thr kinase mTOR is a key integrator of growth-factor-activated and nutrient-sensing pathways in the cell. It is activated by Akt/PKB, which phosphorylates tuberin (TSC2) at multiple sites relieving the inhibitory effects of the hamartin-tuberin (TSC1-TSC2) complex on Rheb and mTOR complex 1 (mTORC1) [52–54]. Activated mTORC1 phosphorylates ribosomal S6 kinase (S6K1), which phosphorylates S6RP [54]. Thus, the increased phosphorylation of S6RP on Ser^235/236^, as seen only in A1-overexpressing cells at high [Mg^2+^]_e_, indicates the increased activity of mTOR (Figure 5C). In our previous work, we have shown that the presence of high [Mg^2+^]_e_ (10 mM) is potent enough to revert the mode of A1 operation from Mg^2+^-extrusion to Mg^2+^-influx leading to a significant increase of [Mg^2+^]_i_ in A1-overexpressing cells [4–6]. Therefore, both the increase of [Mg^2+^]_i_ and the increased levels of A1 expression can be assumed to be necessary to stimulate mTOR signaling. In contrast, the overexpression of A1 paralleled with the presence of no Mg^2+^ or physiological [Mg^2+^] (1 mM) in the bath solution significantly attenuates the phosphorylation of Thr^308^ and Ser^473^ of Akt/PKB in the +tet cells (Figures 5A, 5B, 8A, 8B, Supplementary Figure 3), and, hence, under this condition, the attenuated Akt/PKB activity leads to the increased stability of the TSC1-TSC2 complex and the silencing of mTOR signaling (when compared with [Mg^2+^](10 mM); Figure 5A, 5B, 5D) [52]. Rubin has proposed mTOR as a key player in his Membrane-Magnesium-Mitosis (MMM) model, which holds that any rise in [Mg^2+^]_i_ increases MgATP^2-^ and activates mTOR [55]. Whereas most of the kinases in the PI3K - Akt/PKB – mTOR signaling cascade have a low K_m_ for MgATP^2-^, mTOR has a high requirement that approximates the level of free Mg^2+^ in the cell [55]. Furthermore, the intracellular levels of free Mg^2+^ might play a crucial role in the regulation of the activities of the principal mTOR modulators such as Rheb and Rag GTPases [56–58]. Thus, A1 (its functional and expression status) as a prominent regulator of [Mg^2+^]_i_ and of Akt/PKB activity is a factor of paramount importance in the MMM model.

Perturbed Akt/PKB signaling has been implicated in several serious ailments, e.g., peripheral INS resistance and *diabetes*, Alzheimer's and Parkinson's diseases (AD, PD), and cancer [59–61]. In particular, in cancer, the increased activity of Akt/PKB is considered as being a major factor defining the invasive abilities of tumors [61]. For example, the constitutive phosphorylation of Akt/PKB, particularly on Ser^473^, is associated with poor prognosis in acute myeloid leukemia [62]. Increased Akt/PKB expression and activity have been detected in aggressive human gastric cancers and in breast, prostate, ovarian, and brain tumors [63]. Thus, an understanding of the modalities influencing Akt/PKB activity is absolutely essential for the effective targeting and manipulation of Akt/PKB activity in the aforementioned diseases.

Hypomagnesemia and intracellular Mg^2+^ deficiency are the hallmarks of diseases such as diabetes, AD, or PD and are assumed negatively to influence the course of these diseases and therapeutic outcomes [1, 11]. In contrast, cancer cells are known to retain higher Mg^2+^ concentrations, in agreement with the pro-proliferative effect of Mg^2+^ [34, 64]. Although the identity of NME was unknown at the time, Wolf and Cittadini predicted as early as 1999 that, in tumor cells, the disturbed Mg^2+^ content is probably attributable to the inhibition of Mg efflux via the Na^+^/Mg^2+^ antiporter [64]. Mastrototaro et al. have recently demonstrated that the INS-activated IRTK – PI3K – Akt/PKB signaling axis constitutes an important mechanism controlling the activity of NME A1 [13]. Our present work not only complements the work of Wolf and Cittadini and of Mastrototaro et al., but also most importantly demonstrates that the mutual control of the functions of Akt/PKB (and of Erk1/2 and mTOR) and A1 is delicately balanced, and that the deregulation of either one might result in disease.

Our results also provide support for the role of IMH and the genetics of its molecular constituents in the pathophysiology of prominent human ailments such as cancer. Various aberrant ion-transporting proteins (channels, pumps, and carriers) or the deranged expression of their normal (healthy) variants have been experimentally linked to the process of tumorigenesis (tumor development and progression) [67]. However, the involvement of NME in tumorigenesis has only been hypothesized [64]. Our data provide a solid piece of evidence that NME A1 influences the Akt/PKB signaling node, which thus might interfere with molecular mechanisms discriminating between pro-apoptotic and pro-survival cellular events. In the future, the correlation of the transcriptional and functional parameters of NME A1 and Akt/PKB signaling in cancer and also the other disease conditions mentioned above will be of great interest.

We conclude that the expression status of A1 and the status of [Mg^2+^]_e_ (and consequently also of [Mg^2+^]_i_) play a role in the regulation of Akt/PKB and Erk1/2 activities and the complex physiological response to extracellular stimuli. The levels of expression of *A1* might be considered as a molecular marker in disease conditions hallmarked by disrupted Akt/PKB signaling and/or disturbed IMH. Furthermore, the pharmacological management of the expression and function of A1 might find its place in the therapy of “Akt-opathies”.

## MATERIALS AND METHODS

### Cloning of SLC41A1 into pcDNA5/TO

The coding sequence of SLC41A1 with an N-terminal HA- (hemagglutinin) tag and a C-terminal strep-tag was produced by gene synthesis (ShineGene Bio-Technologies, Inc.) and cloned into pcDNA5/TO via the restriction sites *Kpn*I and *Xho*I.

### Cell culture

HEK293(tet)↑(FLAG-SLC41A1); stable tetracycline-(tet)-inducible HEK293 cells overexpressing N-terminal FLAG-tagged Na^+^/Mg^2+^ exchanger A1. Cells were cultured as previously described by Kolisek and colleagues [4, 5]. Protein expression was induced by the addition of tetracycline (1 μg/mL) for 15 hours. The detailed characteristics of A1 expression in HEK293(tet)↑(FLAG-SLC41A1) cells have been published previously [4, 5]. No leakiness of the expression of recombinant SLC41A1 was detected in –tet cells (Supplementary Figure 11).

The T-REx™-HeLa cell line was purchased from Thermo Fisher Scientific and grown in DMEM (Biochrom) supplemented with 10% FBS and stable glutamine, 1% Pen-Strep, and 5 μg/ml blasticidin.

The human neuroblastoma cell line SH-SY5Y (ATCC CRL-2266) was cultured in Dulbecco's MEM/Ham's F-12 (1:1) (Biochrom) supplemented with 10% FBS and stable glutamine and 1% Pen-Strep.

### Transient transfection of SH-SY5Y and HeLa cells

SH-SY5Y and HeLa cells were transiently transfected with pcDNA5/TO-SLC41A1 or the respective empty control vector by using TurboFect transfection reagent (Thermo Fisher Scientific). For SH-SY5Y, three million cells were seeded per T-75 flask and grown overnight. Subsequently, cells were transiently transfected with the empty vector or the vector harboring SLC41A1 and grown for a further 24 hours. Expression was verified by WB (Supplementary Figure 11).

T-REx™-HeLa cells stably expressing the Tet-repressor were seeded at a density of two million cells per T-75 flask and grown for 24 hours prior transfection. Cells were then transiently transfected and allowed to recover for further 24 hours. Finally, protein expression was induced by the addition of tetracycline (1 μg/mL) for 24 hours. Control cells transfected with pcDNA5/TO-SLC41A1 remained uninduced. Efficient expression of A1 in transiently transfected cells was controlled by immunoblotting with a primary mouse antibody directed against the Strep-tag (1:2,500, Qiagen) in combination with a horseradish peroxidase (HRP)-conjugated secondary antibody (anti-mouse, 1:2,000; from Cell Signaling Technology). No background expression in the absence of tetracycline was detected in HeLa cells as confirmed by immunoblotting (Supplementary Figure 11).

### Cell preparation for label-free assays

HEK293(tet)↑(FLAG-SLC41A1) cells were harvested at 70-80% confluency and seeded into label-free fibronectin-coated microplates (PerkinElmer) in serum-containing medium at a density of 20,000 cells/well. Post-seeding, all cells were incubated at room temperature for 30 minutes prior to overnight incubation under 5% CO_2_ at 37°C. To induce protein expression, cells were incubated, as mentioned above, in label-free microplates.

### Dynamic mass redistribution assay and imaging

Cells were washed three times in 25 μL/well assay buffer (Ca^2+^- and Mg^2+^-free Hank's balanced saline (CMF-HBSS, Biochrom) plus 10 mM HEPES pH 7.4 (CMF-HBSS+)). Washing was carried out by using an aspiration wand and a multichannel pipette. The final volume was 30 μL/well, and potential air bubbles were removed by centrifugation of the plate (400 rpm x 1 min). The label-free microplates were equilibrated for 1 hour at 37°C in an EnSight Multimode Plate Reader (PerkinElmer). DMR signals were measured by using the EnSight Multimode Plate Reader equipped with Corning Epic® label-free technology (http://www.perkinelmer.com/pages/020/labelfree/default.xhtml). An initial baseline measurement was taken for 30 min. The addition of SFLLR-NH_2_ (5 μM), of Mg^2+^ (1, 3, or 10 mM), of Mg^2+^ (1, 3, or 10 mM) and INS (400 μU/mL), or of assay buffer only was followed by a 36-min kinetic measurement. During the whole measurement, the EnSight Multimode Plate Reader was kept at a stable temperature of 37°C. The kinetic data were referenced to the last time point of the baseline measurement.

Brightfield images were acquired by using the well-imaging module of the EnSight Multimode Plate Reader and were taken before the addition of the particular effector, but immediately after the label-free measurement and 90 min after the end of the measurement to allow for a cell confluence comparison over the duration of the experiment. Cell confluence was determined by using the pre-defined Brightfield Confluence analysis method with Kaleido Data Acquisition and Analysis Software (PerkinElmer) as provided by the plate reader.

### PathScan^®^ RTK signaling antibody array-(PRSA)-based investigation of the phosphorylation level of kinases and key signaling nodes in –tet and +tet cells

Four individual aliquots of –tet and +tet HEK293(tet)↑(FLAG-SLC41A1) cells were washed once with completely divalent-cation-free Dulbecco's phosphate-buffered saline (DPBS) and subsequently with CMF-HBSS+. After these washing steps, the first aliquots of –tet and +tet cells were directly lysed (C0) in 1x Cell Lysis Buffer (New England Biolabs), whereas the other aliquots were incubated in CMF-HBSS+ for 20 min at 37°C (C1). Cells were subsequently pelleted and resuspended in CMF-HBSS+ plus 10 mM MgCl_2_ and incubated for 30 min at 37°C (C2). Finally, cells were transferred to CMF-HBSS+ supplemented with 400 μU/mL INS and incubated for another 25 min (C3). After each change of the incubation conditions, one aliquot of –tet and +tet cells was lysed in 1x Cell Lysis Buffer. Unsolubilized material was pelleted by centrifugation (14,000 rpm, 10 min, 4°C). The protein concentration of the sample was determined with the Pierce 660 nm protein assay (Thermo Fisher Scientific), and lysates were diluted to a protein concentration of 0.8 mg/mL. The PRSA Kit (New England Biolabs) was used according to the manufacturer's instructions. Cell lysates were incubated on slides overnight at 4°C. The chemiluminescence detection of the spots was performed with the ChemiDoc MP imaging system (Bio-Rad), and spot intensities were quantified with the inbuilt software Image Lab^TM^ 5.2.1 (Bio-Rad). The presented relative densities (RD) were calculated from the pixel densities of particular phospho-signals normalized to the pixel densities of positive control spots printed on the chip.

### Determination of phosphorylation status of Akt/PKB in response to overexpression of A1

For the WB detection of Ser^473^- and Thr^308^-phosphorylated Akt, –tet and +tet cells were treated exactly as described above for PRSA. Unsolubilized material was pelleted by centrifugation (14,000 rpm, 10 min, 4°C), and the protein concentration was determined by the Pierce 660 nm protein assay (Thermo Fisher Scientific). For HEK293(tet)↑(FLAG-SLC41A1) cells, 30 μg total protein was used for immunoblotting. The same protein amount was used for immunodetection with HeLa cells. For the detection of the phosphorylation status of Akt/PKB-Thr^308^ or Akt/PKB -Ser^473^ in SH-SY5Y, 20 μg total protein were loaded for conditions C0-C2 and 5 μg for C3. For the detection of Erk1/2-Thr^202^/Tyr^204^ or total Erk1/2 in SH-SY5Y, 20 μg of total protein was used for all conditions. Protein samples were run on 10% SDS-PAA gels and transferred to polyvinylidene difluoride (PVDF) membranes. For immunodetection, (1) monoclonal rabbit antibodies recognizing either Akt/PKB-Thr^308^ or Akt/PKB -Ser^473^ (Phospo-Akt Thr^308^ clone D25E6, and Phospo-Akt Ser^473^ clone D9E; both from New England Biolabs) diluted (1:800 and 1:1,000, respectively) in 2.5% milk TBS-T (Tris-buffered saline and Tween 20) were used in combination with a horseradish peroxidase (HRP)-conjugated secondary anti-rabbit antibody (1:2,500, Promega) or (2) polyclonal rabbit antibodies recognizing either Erk1/2-Thr^202^/Tyr^204^ or total Erk1/2 (both from New England Biolabs) diluted (both 1:1,000) in 2.5% milk TBS-T were used in combination with a HRP-conjugated secondary anti-rabbit antibody (1:2,500, Promega). An antibody against RPL19 (Abnova), together with an HRP-coupled anti-mouse secondary antibody (Promega), was used to detect the loading control. Proteins were visualized with the Clarity™ ECL Western Blotting Substrate (Bio-Rad). Image J software (http://rsb.info.nih.gov/ij/) and Image Lab^TM^ 5.2.1 software (BioRad) were used for the densitometric analysis of WB.

Relative adjusted densities (RAD) were calculated from the relative densities (RD) of Akt/PKB-Thr^308^ or Akt/PKB-Ser^473^ and of RPL19 bands (RAD = RD_Akt/PKB_ /RD_RPL19_).

RAD were calculated for Erk1/2 from RD of phosphorylated Erk1/2-Thr^202^/Tyr^204^ and of total Erk1/2 bands (RAD = RD_Erk1/2-Thr202/Tyr204_/RD_Erk1/2(total)_).

RD were calculated as the pixel density (D) of the particular –tet and +tet bands obtained after treatment C0, C1, C2, or C3 divided by D of reference bands (corresponding to those obtained after treatment C0 in –tet cells).

Due to inconsistencies in membrane stripping in SH-SY5Y and HeLa cells we decided for total protein staining as normalization method for the detection of Erk1/2-Thr^202^/Tyr^204^ phosphorylation in these cell lines. The TGX Stain-Free^TM^ FastCast^TM^ Kit (Bio-Rad) was used to prepare 10% SDS-PAA gels. Protein detection with this technique is based on trihalo-compound modification of tryptophan residues and has proven to be superior to the conventional detection of housekeeping-proteins [68–70]. Loaded protein amounts were as described previously and gels for the detection of total and phosphorylated Erk1/2 were run in parallel. Prior to immunoblotting the stain-free gels were UV-activated for 1 min and pictures were taken using the ChemiDoc MP imaging system (Bio-Rad, Supplementary Figure 12A, Supplementary Figure 13A, Supplementary Figure 14A, Supplementary Figure 15A). Proteins were transferred to PVDF membranes and proteins on the membrane were imaged and quantified using Image Lab^TM^ 5.2.1 software (Supplementary Figure 12B, Supplementary Figure 13B, Supplementary Figure 14B, Supplementary Figure 15B).

RD for total Erk1/2 and phosphorylated Erk1/2-Thr^202^/Tyr^204^ was first normalized on total protein (RD_Erk1/2(total)_/RD_total protein_ = RDN_Erk1/2(total)_) and RD_Erk1/2-Thr202/Tyr204_/RD_total protein_ = RDN_Erk1/2-Thr202/Tyr204_) and RAD were calculated from the normalized relative densities (RDN) of phosphorylated Erk1/2-Thr^202^/Tyr^204^ and normalized total Erk1/2 (RAD = RDN_Erk1/2-Thr202/Tyr204_ /RDN_Erk1/2(total)_).

### Mag-fura 2 assisted Mg^2+^-measurements

The −tet and +tet HEK293(tet)↑(FLAG-SLC41A1) cells were rinsed twice with ice-cold, completely divalent, cation-free DPBS, detached by HyQtase (HyClone), centrifuged, washed in CMF-HBSS+, and resuspended in HBSS+ supplemented with [Mg^2+^] = 1 mM. Loading of cells with mag-fura 2 AM were performed as following: (C0) 30-min loading with mag-fura 2 in presence of [Mg^2+^]_e_ = 1 mM. [Mg^2+^]_i_ were measured in –tet and +tet cells immediately after a 30-min loading period; (C1) 30-min loading with mag-fura 2 in CMF-HBSS+. [Mg^2+^]_i_ were measured in –tet and +tet cells immediately after a 30-min loading period. Notably, the duration of the C1 treatment for mag-fura 2-assisted [Mg^2+^]_i_ measurements was extended by 10 min when compared with the duration of the C1 treatment in phosphorylation experiments. This extension was necessary to allow for the sufficient mag-fura 2 AM loading into cells and its activation (de-esterification) by intracellular esterases; (C2) 30-min loading with mag-fura 2 in CMF-HBSS+ plus 30-min incubation of the cells in HBSS+ containing 10 mM Mg^2+^. [Mg^2+^]_i_ were measured in –tet and +tet cells immediately after 30-min Mg^2+^ loading period; (C3) 30-min loading with mag-fura 2 in Mg^2+^-free HBSS+ plus 30 min incubation of the cells in HBSS+ containing 10 mM Mg^2+^ and followed by 25-min incubation of the cells in CMF-HBSS+ provided with INS (400 μU/mL, measuring solution). The following [Mg^2+^]_e_ were present in the measuring media under the respective conditions: C0, 1 mM; C1, 0.00 mM; C2, 10 mM; C3, 0.00 mM. Moreover, under C3, INS (400 μU/mL) was added to the measuring medium. Measurements were executed at 37°C in 3-ml cuvettes containing 2 ml cell suspension while being stirred. [Mg^2+^]_i_ were determined by measuring the fluorescence of the probe-loaded cells in a spectrometer (LS50B and LS55, both PerkinElmer Life Science) by using the fast-filter accessory (340/380 nm; PerkinElmer Life Science). [Mg^2+^]_i_ values were calculated according to previous reports 10,13,20. Minimum (Rmin) and maximum (Rmax) ratios for calibration were determined at the end of each experiment by using digitonin (SigmaAldrich). R_max_ was found by the addition of MgCl_2_ to its final concentration of 25 mM in the absence of Ca^2+^, whereas R_min_ was obtained by the addition of 50 mM EDTA, pH 7.2 (SigmaAldrich). Averaged values of [Mg^2+^]_i_ were calculated from 20-s data sets. The effective frequency of data acquisition was 20 ms. Thus, for the calculation of any given averaged [Mg^2+^]_i_, 1000 data points were used. Measurements for each respective group were acquired at least on three separate occasions.

### Statistics

(1) The two-tailed Student's t-test was used to compare differences between two means (Tables 1 and 2, Figures 5, 8, 10, Supplementary Figure 3, Supplementary Figure 5, Supplementary Figure 7, Supplementary Figure 9). (2) A *post hoc* Tukey one-way analysis of variance (ANOVA) or Kruskal–Wallis one-way ANOVA on ranks was used to perform a multiple comparison between treatment groups (C0, C1, C2, and C3) in the –tet or +tet groups (Table 3, Figures 6, 8, 10, Supplementary Figures 3, 5, 9). A Shapiro-Wilk normality test was used for (2). Data are presented as means ± SE. Differences of P < 0.05 were considered significant. Statistical analyses were executed by means of SigmaPlot 11.0 (Systat Software, Inc.). The same software was used to generate graphs.

## SUPPLEMENTARY MATERIALS FIGURES



## Abbreviations

AD: Alzheimer's disease
Akt/PKB: protein kinase B
ANG: angiotensin II
AR: androgen receptor
*ARE*s: androgen-responsive elements
CMF-HBSS: Ca^2+^- and Mg^2+^-free Hank's balanced salt solution
CMF-HBSS+: Ca^2+^- and Mg^2+^-free Hank's balanced salt solution plus 10 mM HEPES pH 7.4
c-Src (Src): proto-oncogene tyrosine-protein kinase Src
D: pixel density
DMR: dynamic mass redistribution
DPBS: Dulbecco's phosphate-buffered saline
EGF: epidermal growth factor
EGFR: epidermal growth factor receptor
Erk1/2: extracellular signal-regulated kinase 1/2
FAK: focal adhesion kinase
GβL: G protein beta subunit like
GH: growth hormone
GHR: growth hormone receptor
IMD: intracellular Mg^2+^ deficiency
IMH: intracellular magnesium homeostasis
INFγ: interferon-gamma
INFγR: interferon-gamma receptor
INS: insulin
IGF-1: insulin-like growth factor 1
IRS1 and IRS2: insulin receptor substrate 1 and 2
IRTK: insulin receptor tyrosine kinase
JAK2: Janus kinase 2
L: leptin
LIF: leukemia inhibitory factor
LIFR: leukemia inhibitory factor receptor
mTOR: mechanistic target of rapamycin
mTORC1: mechanistic target of rapamycin complex 1
NME: Na^+^/Mg^2+^ exchanger
PAR-1: protease-activated receptor 1
PD: Parkinson's disease
PDE3b: phosphodiesterase 3b
PDGF: platelet-derived growth factor
PDGFR: platelet-derived growth factor receptor
PDK-1: 3-phosphoinositide dependent protein kinase-1
PHLPP: PH domain and leucine-rich repeat protein phosphatase 1
PI3K: phosphatidylinositol-4,5-bisphosphate 3-kinase
PKA: protein kinase A
PP2A: protein phosphatase 2
PRSA: PathScan^®^ RTK Signaling Antibody Array
PTPA: protein tyrosine phosphatase
RAD: relative adjusted density
RD: relative density
RICTOR: rapamycin-insensitive companion of mTOR
RPL19: 60S ribosomal protein L19
RTK: receptor tyrosine kinase
S6K1: ribosomal S6 kinase
S6RP: S6 ribosomal protein
SFLLR-NH_2_: protease-activated receptor 1 activating peptide
SLC41A1: solute carrier family 41 member A1
TBS-T: Tris-buffered saline and Tween 20
tet: tetracycline
TSC1: hamartin
TSC2: tuberin
TSC1-TSC2: hamartin-tuberin complex
WB: Western blot
