# Supplementary material for: Overexpression of Na+/Mg2+ exchanger SLC41A1 attenuates pro-survival signaling
Source: Oncotarget. 2017 Dec 22;9(4):5084–104. doi: 10.18632/oncotarget.23598 (PMC5797035; doi:10.18632/oncotarget.23598)
Supplement: Supplementary file 1 [file oncotarget-09-5084-s001.pdf]

# Overexpression of $Na^{+}/Mg^{2+}$ exchanger SLC41A1 attenuates pro-survival signaling

## SUPPLEMENTARY MATERIALS

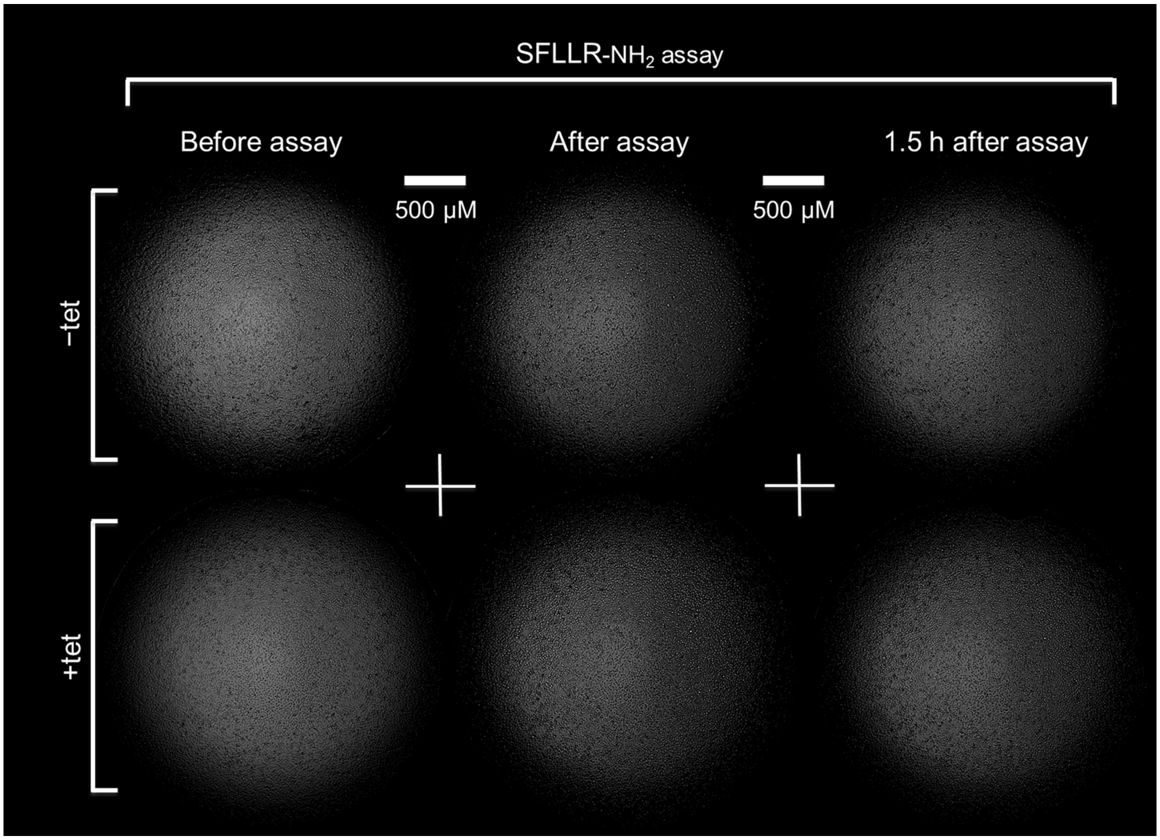


**Supplementary Figure 1: Brightfield images of –tet and +tet cells acquired by using the well-imaging module of the EnSight Multimode Plate Reader taken before the addition of SFLLR-$NH_2$, immediately after the label-free DMR measurement, and 1. 5 hrs after the end of the measurement.** Cell confluence was determined by using the pre-defined Brightfield Confluence analysis method with Kaleido Data Acquisition and Analysis Software (PerkinElmer). Abbreviations: SFLLR-$NH_2$, protease-activated receptor 1 activating peptide; tet, tetracycline.

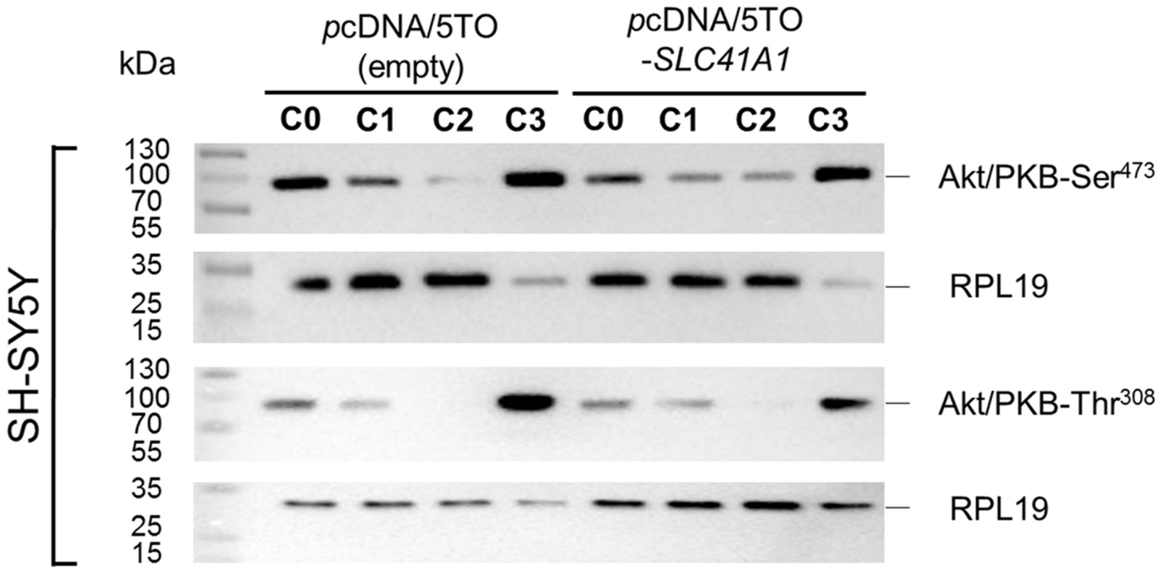


**Supplementary Figure 2: WB analysis of the phosphorylation status of Akt/PKB-Thr[308] and Akt/PKB- Ser[473] in response to the treatment conditions C0, C1, C2, C3 (see Figure 4 in the main document for details) in SH-SY5Y cells transfected with empty *p*cDNA/5TO (empty) or with *p*cDNA/5TO-*SLC41A1* (overexpressing SLC41A1).** One representative experiment of, in total, six independent biological experiments is shown. RPL19 was used as loading reference and was detected subsequent to the phospho-signals on the same blots. Abbreviations: Akt/PKB, protein kinase B; C, condition; RPL19, 60S ribosomal protein L19; WB, Western blot.

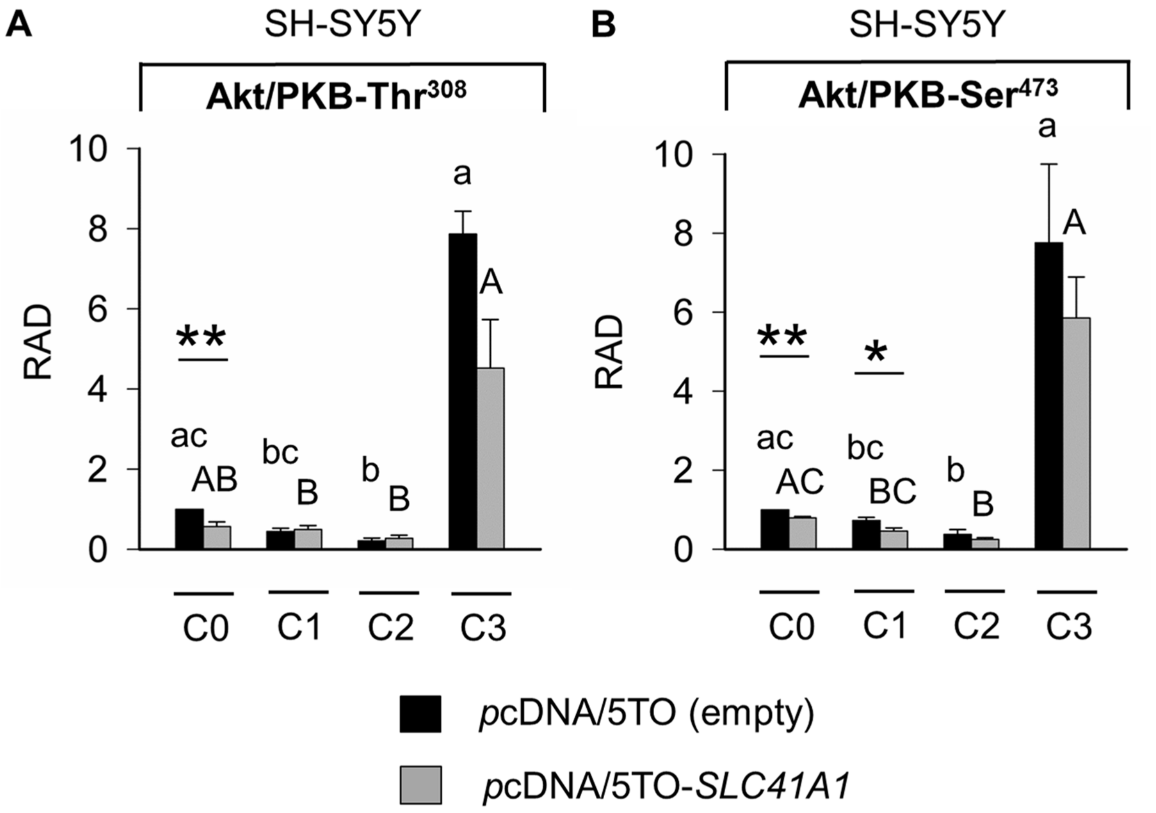


**Supplementary Figure 3:** Pairwise comparison of relative adjusted densities of Akt/PKB-Thr$^{308}$ **(A)** and Akt/PKB-Ser$^{473}$ **(B)** phospho-signals (obtained with WB) and between-treatments (C0, C1, C2, C3; see Figure 4 in the main document for details) comparison of relative adjusted densities of Akt/PKB-Thr$^{308}$ (A) and Akt/PKB-Ser$^{473}$ (B) phospho-signals in SH-SY5Y cells transfected with empty *p*cDNA/5TO or with *p*cDNA/5TO-*SLC41A1* (overexpressing SLC41A1). Data are presented as means (N = 6) ± SE. Pairwise comparisons: Significance is being indicated ($^{*}P < 0.05$, $^{**}P < 0.01$). Between-treatment comparisons: Labeled means without a common letter differ ($P < 0.05$). Small letters were used for the between-treatment comparisons in group "transfected with empty plasmid" and capital letters in group "transfected with *SLC41A1*-containing construct". Please note, for both (A) and (B) between-treatment comparisons conservative *post hoc* Tukeys procedure was applied. If moderately less conservative *post hoc* Student-Newman-Keuls procedure was applied for both (A) and (B), all pairwise comparisons between the treatment groups were found significant with ($P < 0.05$). Abbreviations: Akt/PKB, protein kinase B; C, condition; RAD, relative adjusted density; WB, Western blot.

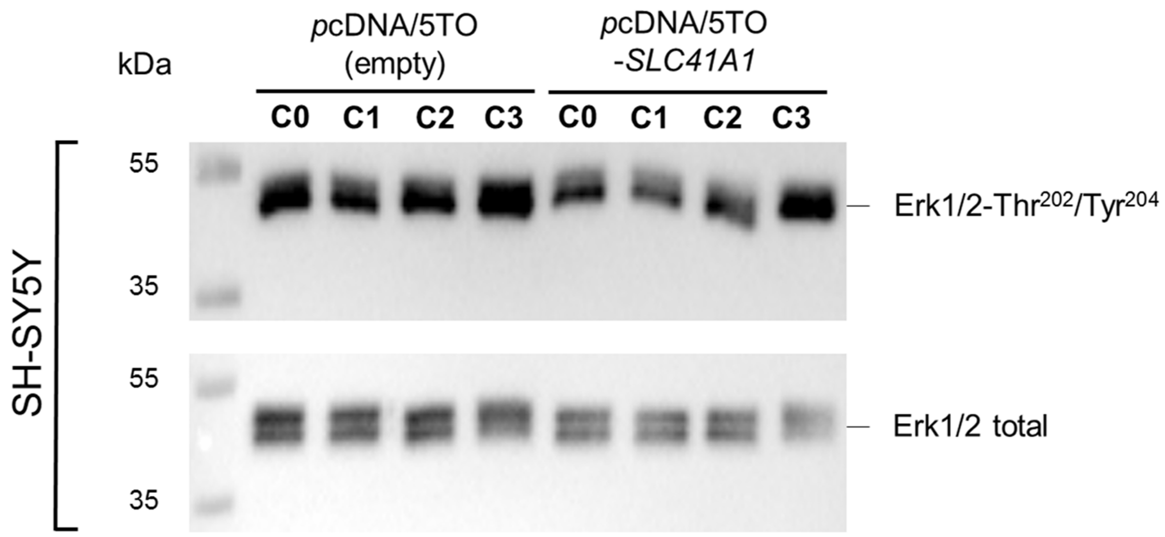


**Supplementary Figure 4: WB analysis of the phosphorylation status of Erk1/2-Thr$^{202}$/Tyr$^{204}$ in response to the treatment conditions C0, C1, C2, C3 (see Figure 4 in the main document for details) in SH-SY5Y cells transfected with empty *p*cDNA/5TO (empty) or with *p*cDNA/5TO-*SLC41A1* (overexpressing SLC41A1).** One representative experiment of, in total, six independent biological experiments in which total Erk1/2 and the phospho-signals of Erk1/2-Thr$^{202}$/Tyr$^{204}$ were detected in parallel. Abbreviations: C, condition; Erk1/2, extracellular signal-regulated kinase 1/2; WB, Western blot.

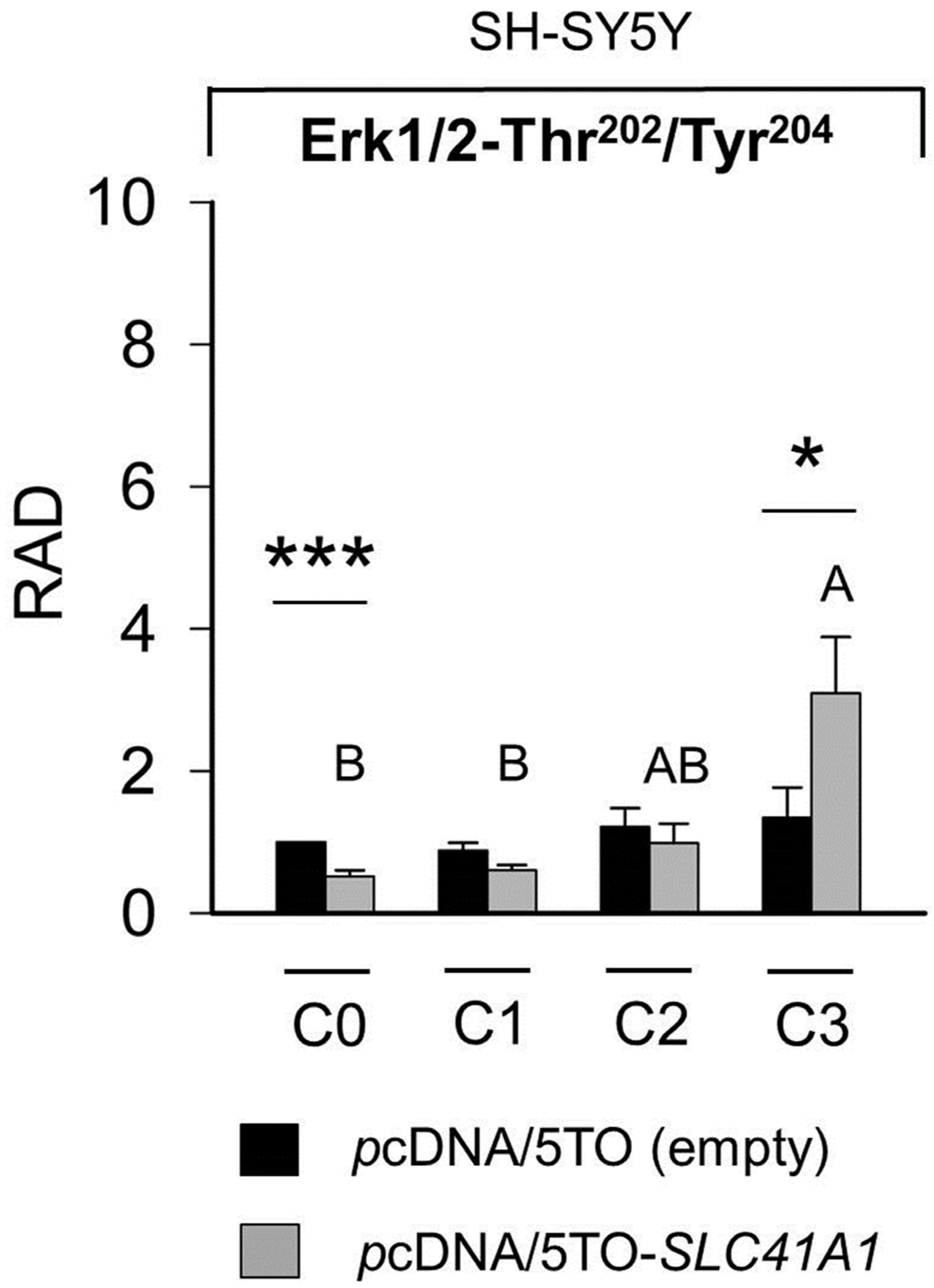


**Supplementary Figure 5: Pairwise comparison of relative adjusted densities of Erk1/2-Thr$^{202}$/Tyr$^{204}$ phospho-signals (obtained with WB) and between-treatments (C0, C1, C2, C3; see Figure 4 in the main document for details) comparison of relative adjusted densities of Erk1/2-Thr$^{202}$/Tyr$^{204}$ phospho-signals in SH-SY5Y cells transfected with empty *p*cDNA/5TO or with *p*cDNA/5TO-*SLC41A1* (overexpressing SLC41A1).** Data are presented as means (N = 6) ± SE. Pairwise comparisons: Significance is being indicated ($^{*}P < 0.05$, $^{***}P < 0.001$). Between-treatment comparisons: Labeled means without a common letter differ ($P < 0.05$). Small letters were used for the between-treatment comparisons in "transfected with empty plasmid" group and capital letters in "transfected with *SLC41A1*-containing construct" group. Abbreviations: C, condition; Erk1/2, extracellular signal-regulated kinase 1/2; RAD, relative adjusted density; WB, Western blot.

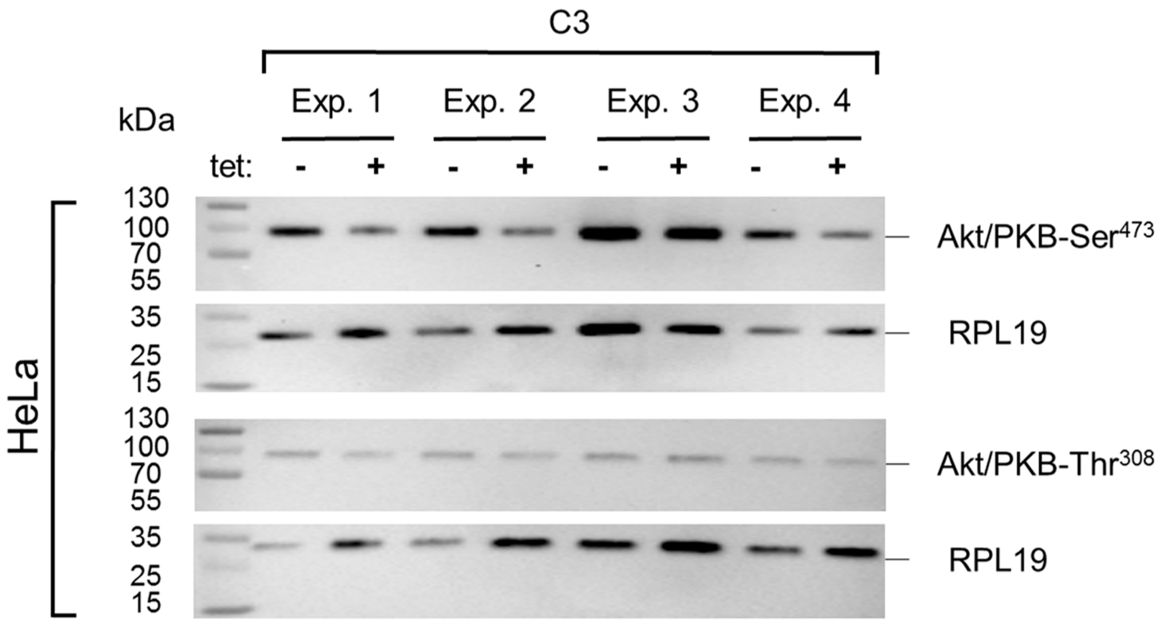


**Supplementary Figure 6: WB analysis of the phosphorylation status of Akt/PKB-Thr$^{308}$ and Akt/PKB- Ser$^{473}$ in response to the treatment conditions C3 (see Figure 4 in the main document for details) in –tet (control) and +tet (SLC41A1-overexpressing) HeLa cells.** Results of four independent biological experiments are shown. RPL19 was used as loading reference and was detected subsequent to the phospho-signals on the same blots. Abbreviations: Akt/PKB, protein kinase B; C, condition; RPL19, 60S ribosomal protein L19; tet, tetracycline; WB, Western blot.

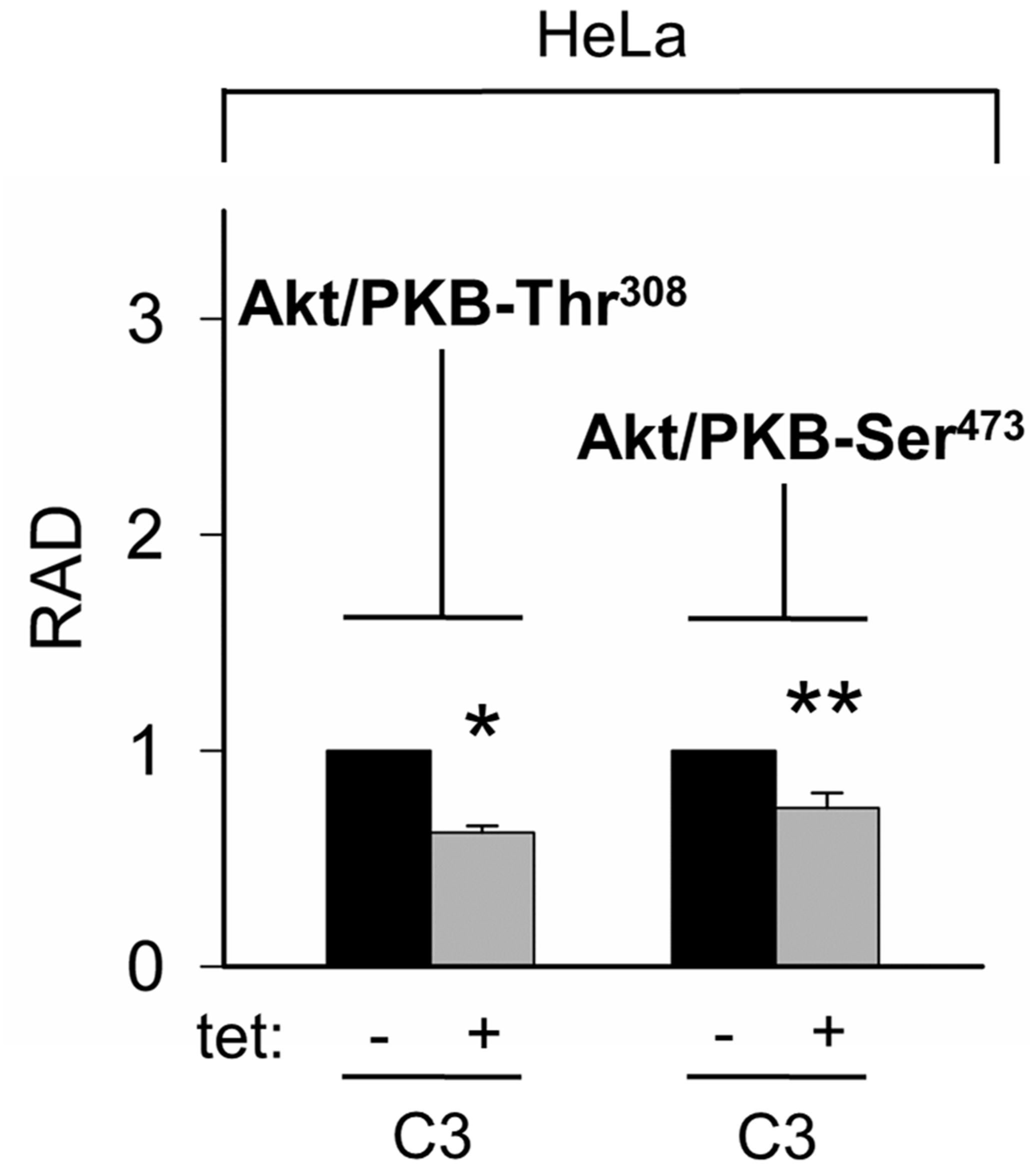


**Supplementary Figure 7: Pairwise comparison of relative adjusted densities of Akt/PKB-Thr$^{308}$ and Akt/PKB-Ser$^{473}$ phospho-signals (obtained with WB) in –tet (control) and +tet (SLC41A1-overexpressing) HeLa cells treated under C3.** Data are presented as means (N = 5) ± SE. Pairwise comparisons: Significance is being indicated ($^{*}P < 0.05$, $^{**}P < 0.01$). Abbreviations: Akt/PKB, protein kinase B; C, condition; RAD, relative adjusted density; tet, tetracycline; WB, Western blot.

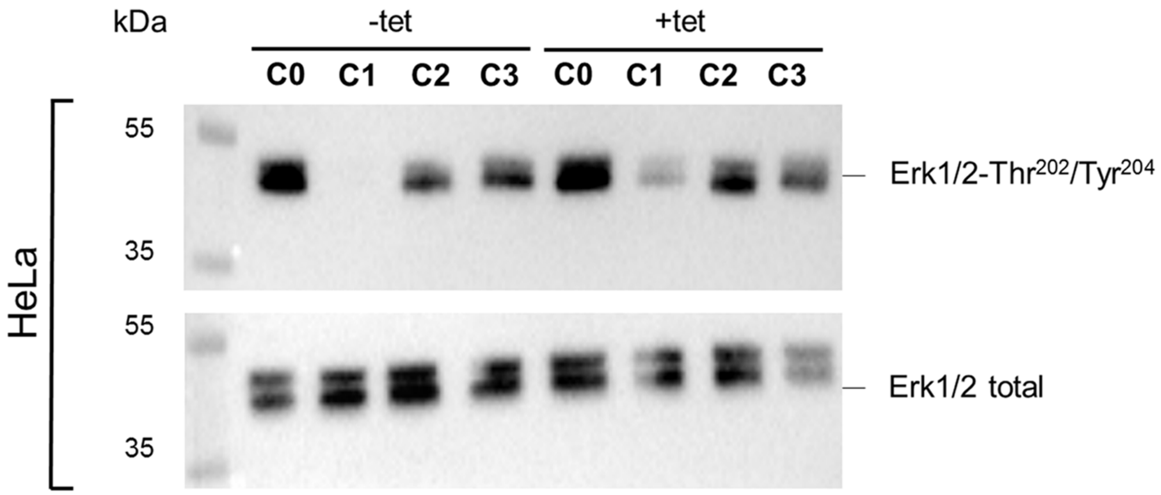


**Supplementary Figure 8: WB analysis of the phosphorylation status of Erk1/2-$Thr^{202}$/$Tyr^{204}$ in response to the treatment conditions C0, C1, C2, C3 (see Figure 4 in the main document for details) in –tet (control) and +tet (SLC41A1-overexpressing) HeLa cells.** One representative experiments of, in total, four independent biological experiments in which total Erk1/2 and the phospho-signals of Erk1/2-$Thr^{202}$/$Tyr^{204}$ were detected in parallel. Abbreviations: C, condition; Erk1/2, extracellular signal-regulated kinase 1/2; tet, tetracycline; WB, Western blot.

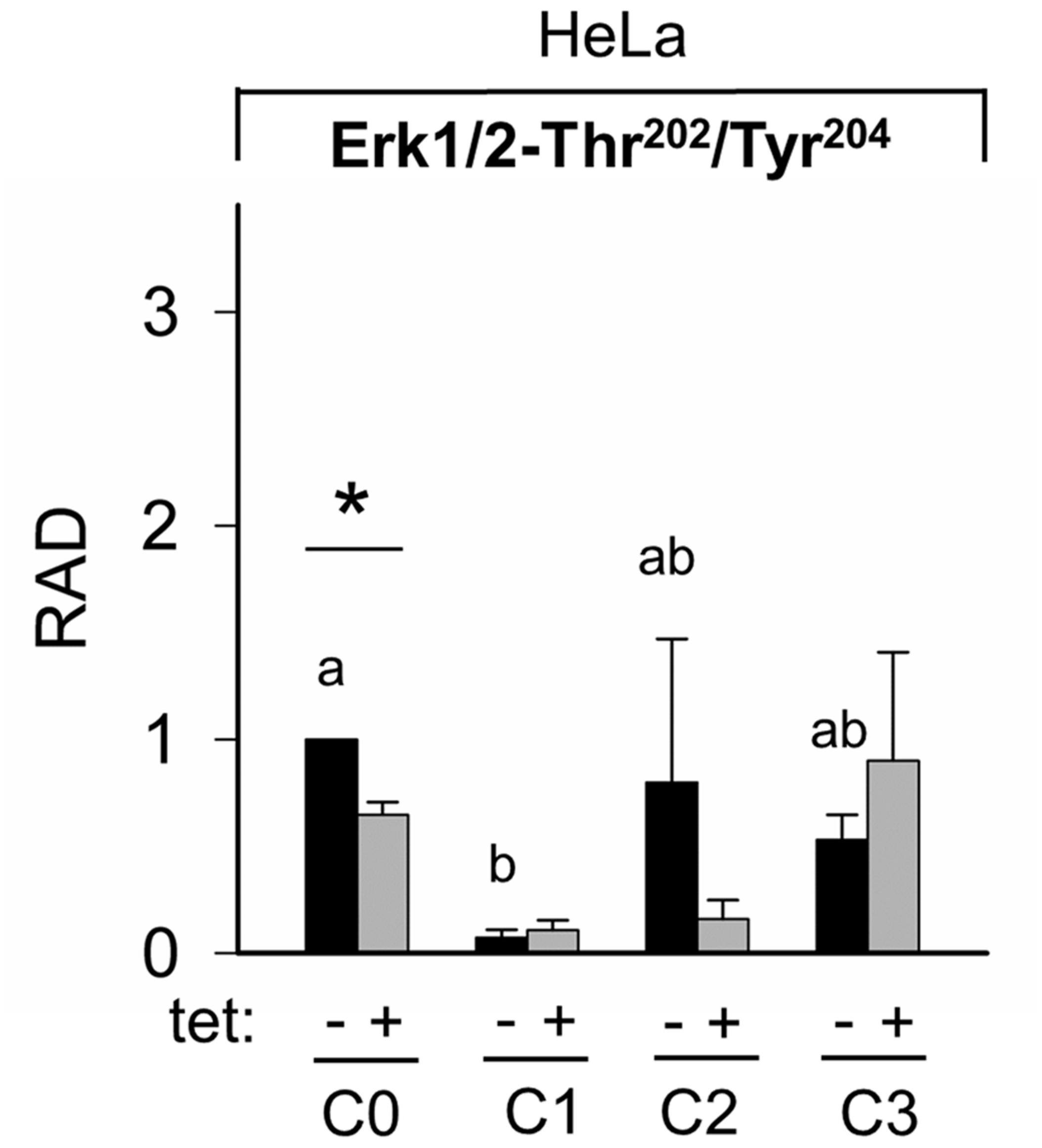


**Supplementary Figure 9: Pairwise comparison of relative adjusted densities of Erk1/2-Thr$^{202}$/Tyr$^{204}$ phospho-signals and between-treatments (C0, C1, C2, C3; see Figure 4 for details) comparison of relative adjusted densities of Erk1/2-Thr$^{202}$/Tyr$^{204}$ phospho-signals in –tet (control) and +tet (SLC41A1-overexpressing) HeLa cells, obtained with WB.** Data are presented as means (N = 4) ± SE. Pairwise comparisons: significance is being indicated ($^*P < 0.05$). Between-treatment comparisons: Labeled means without a common letter differ ($P < 0.05$). Small letters were used for the between-treatment comparisons in –tet group and capital letters in +tet group. Abbreviations: C, condition; Erk1/2, extracellular signal-regulated kinase 1/2; RAD, relative adjusted density; tet, tetracycline; WB, Western blot.

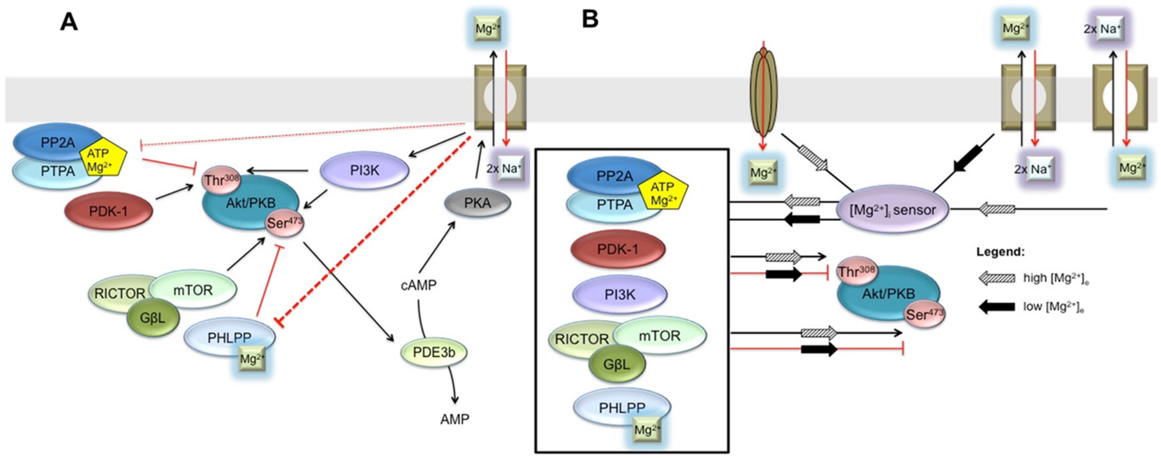


**Supplementary Figure 10: (A)** Expression of recombinant SLC41A1 in –tet and +tet HEK293 cells upon condition: C0, C1, C2 and C3. **(B)** Expression of recombinant SLC41A1 in SH-SY5Y cells transfected with empty *p*cDNA/5TO or with *p*cDNA/5TO-*SLC41A1* (overexpressing SLC41A1). **(C)** Expression of recombinant SLC41A1 in –tet and +tet HeLa cells upon condition: C0, C1, C2 and C3. Abbreviations: C, condition; RPL19, 60S ribosomal protein L19; tet, tetracycline.

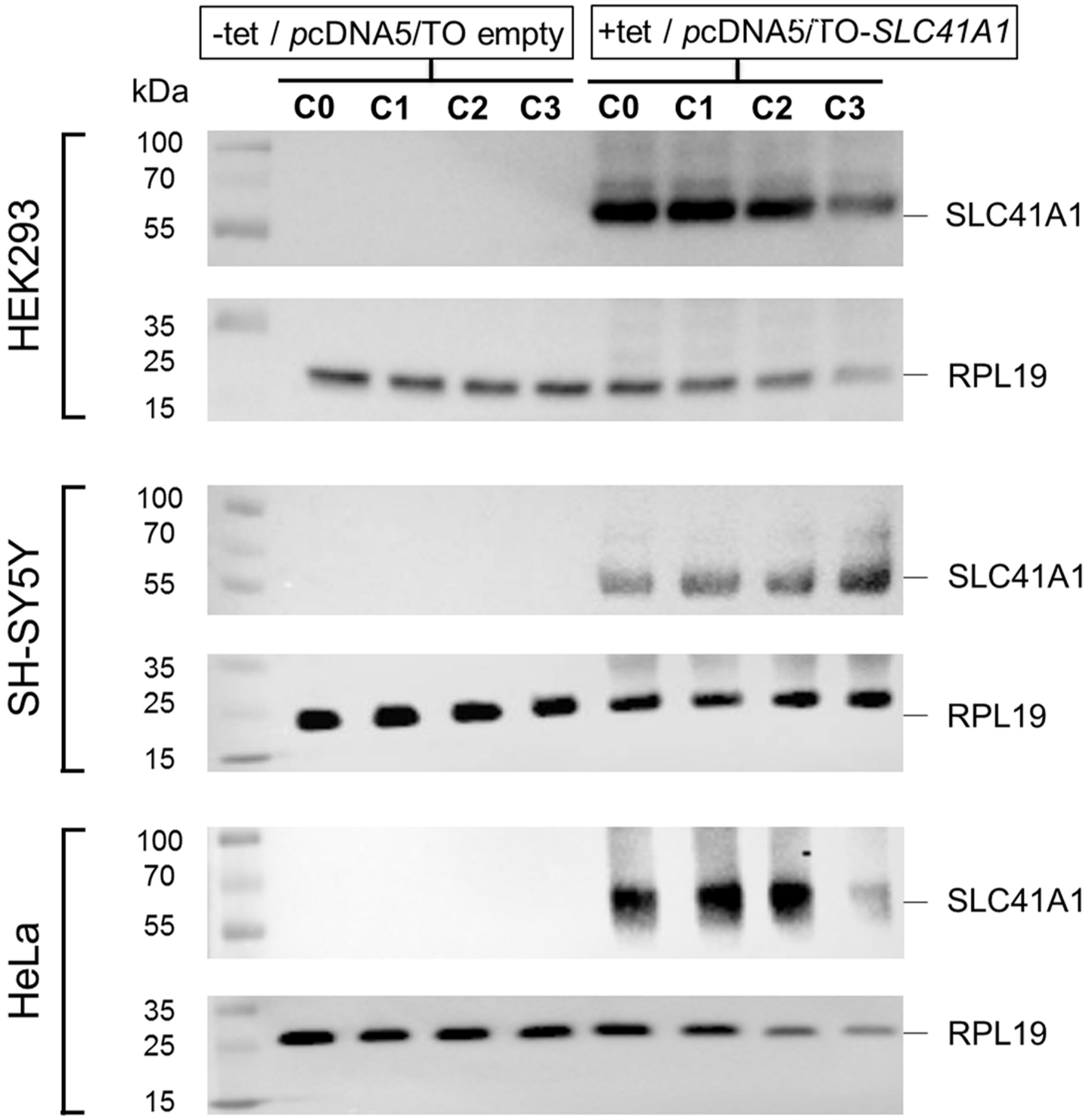


**Supplementary Figure 11:** Hypothetical models of the functional interplay between $Na^+$/$Mg^{2+}$ exchanger SLC41A1 and Akt/PKB: **(A)** without involvement of $Mg^{2+}$-sensor; **(B)** with $Mg^{2+}$-sensor being involved. (A) The putative model depicting the effect of the low $[Mg^{2+}]_i$, resulting from the overexpression and/or deregulated (excessive) A1-dependent $Mg^{2+}$ efflux on the functional down-regulation of PHLPP and PP2A and the activation of PI3K, and thus, on the increased phosphorylation of both Akt/PKB-$Thr^{308}$ and -$Ser^{473}$. The dashed line represents an as yet unconfirmed but highly probable link between the low $[Mg^{2+}]_i$ caused by A1-dependent $Mg^{2+}$ extrusion and inhibition of PHLPP. (B) The more complex, but also more probable, putative model of a functional interaction between A1 and Akt/PKB involving the $[Mg^{2+}]_i$ sensor that adapts Akt/PKB activity (its phosphorylation) in response to A1 expression level, its functional status, and mode of operation ($Mg^{2+}$ influx/efflux). The $Mg^{2+}$ sensor might provide integral information about the status of intracellular $[Mg^{2+}]_i$ to various molecules that consequently influence key cellular processes such as Akt/PKB prosurvival signaling. Abbreviations: Akt/PKB, protein kinase B; GβL, G protein beta subunit like; mTOR, mechanistic target of rapamycin; PDE3b, phosphodiesterase 3b; PDK-1, 3-phosphoinositide dependent protein kinase-1; PI3K, phosphatidylinositol-4,5-bisphosphate 3-kinase; PHLPP, PH domain and leucine-rich repeat protein phosphatase 1; PKA, protein kinase A; PP2A, protein phosphatase 2; PTPA, protein tyrosine phosphatase; RICTOR, rapamycin-insensitive companion of mTOR.

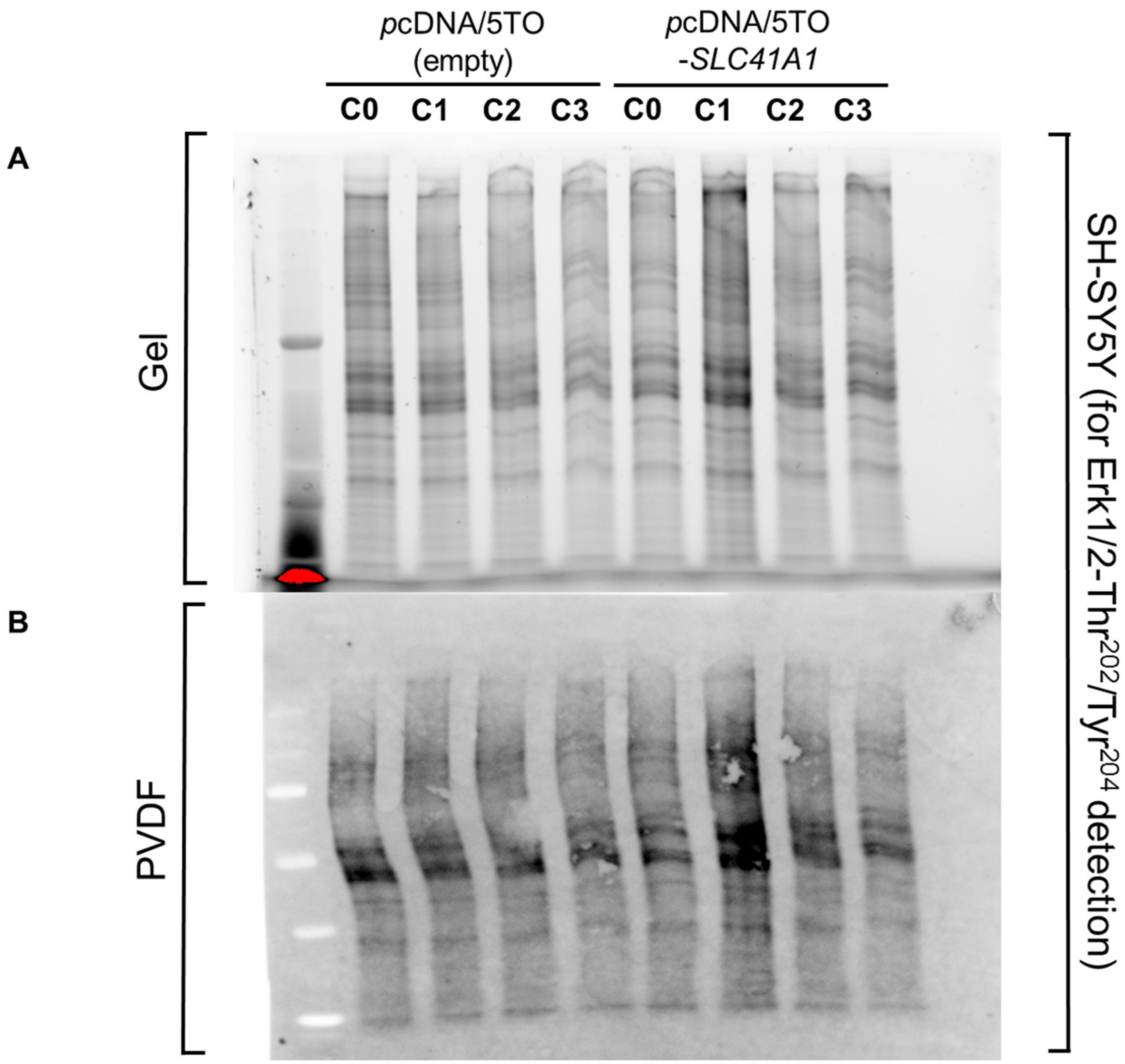


**Supplementary Figure 12: An example of the total protein staining of *p*cDNA/5TO-(empty)-transfected and *p*cDNA/5TO-*SLC41A1*-transfected SH-SY5Y cells.** Figure **(A)** depicts the gel with stained total protein. Figure **(B)** shows the total protein blotted from the gel onto the PVDF membrane. This membrane was used for further WB detection of Erk1/2-$Thr^{202}$/$Tyr^{204}$ shown in SF 4. Abbreviations: C, condition; Erk1/2, extracellular signal-regulated kinase 1/2; PVDF, polyvinylidene difluoride; WB, Western blot.

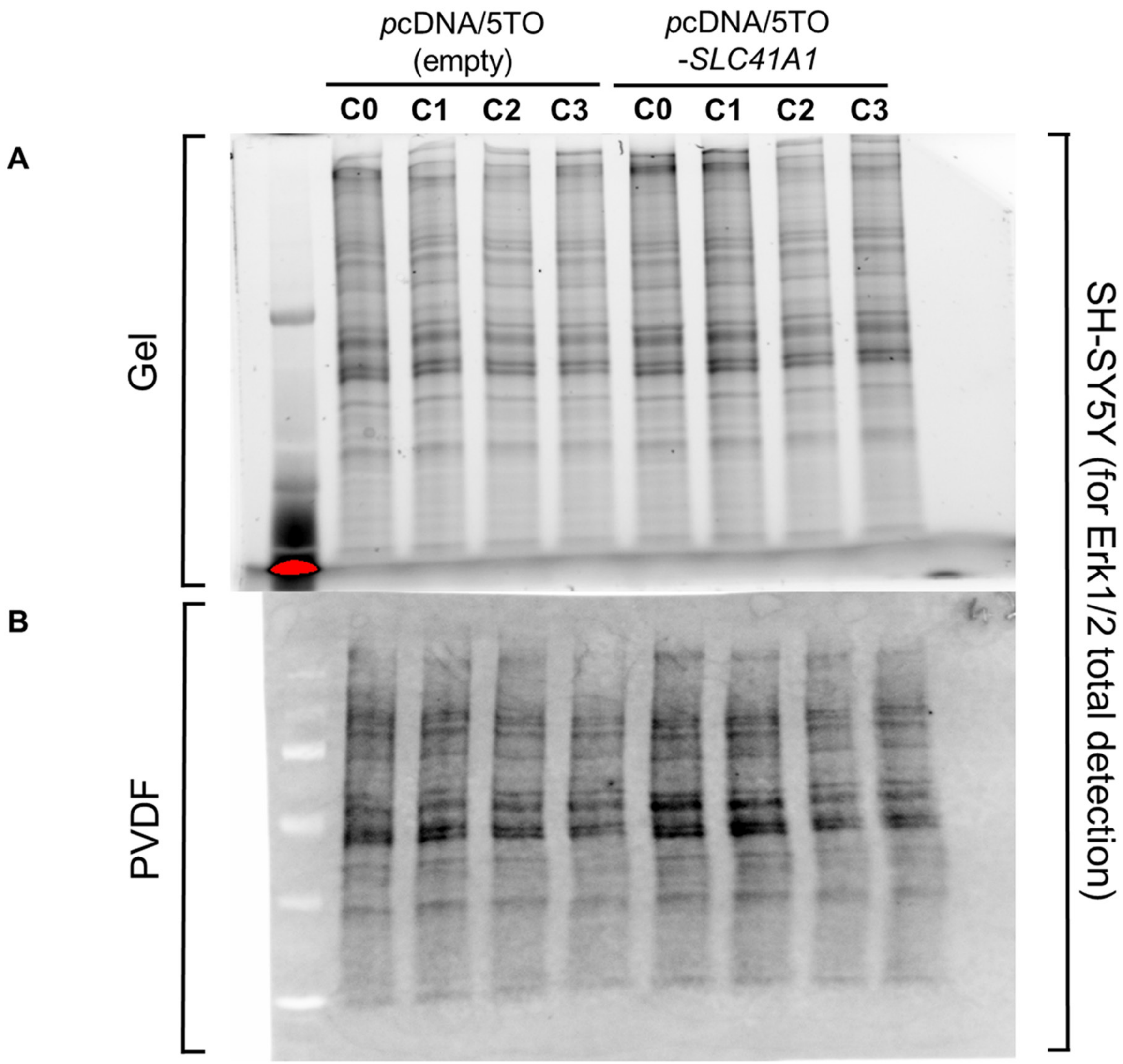


**Supplementary Figure 13: An example of the total protein staining of *p*cDNA/5TO-(empty)-transfected and *p*cDNA/5TO-*SLC41A1*-transfected SH-SY5Y cells.** Figure **(A)** depicts the gel with stained total protein. Figure **(B)** shows the total protein blotted from the gel onto the PVDF membrane. This membrane was used for further WB detection of total Erk1/2 shown in SF 4. Abbreviations: C, condition; Erk1/2, extracellular signal-regulated kinase1/2; PVDF, polyvinylidene difluoride; WB, Western blot.

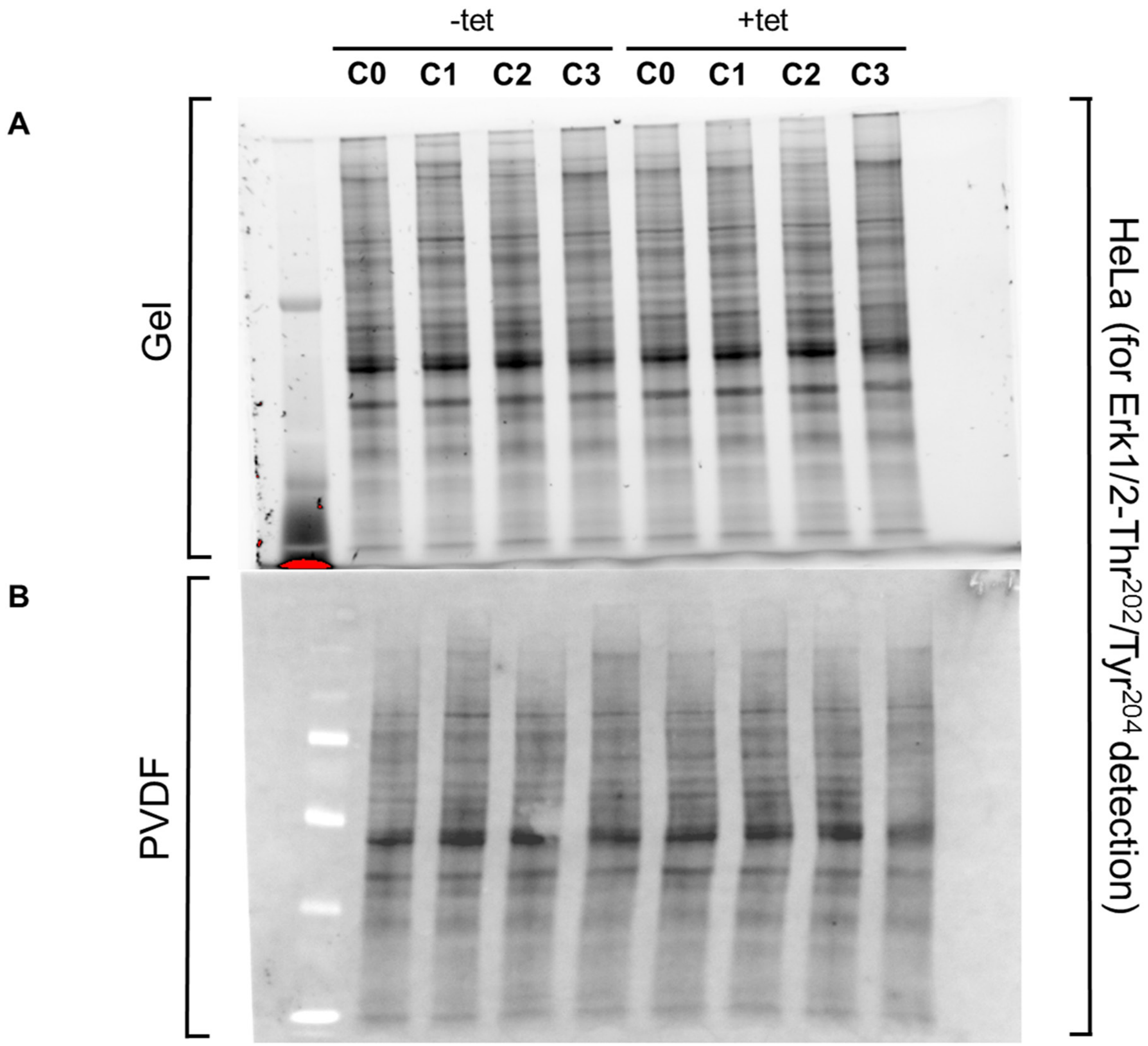


**Supplementary Figure 14: An example of the total protein staining of –tet and +tet HeLa cells.** Figure **(A)** depicts the gel with stained total protein. Figure **(B)** shows the total protein blotted from the gel onto the PVDF membrane. This membrane was used for further WB detection of Erk1/2-Thr$^{202}$/Tyr$^{204}$ shown in SF 8. Abbreviations: C, condition; Erk1/2, extracellular signal-regulated kinase 1/2; PVDF, polyvinylidene difluoride; tet, tetracycline; WB, Western blot.

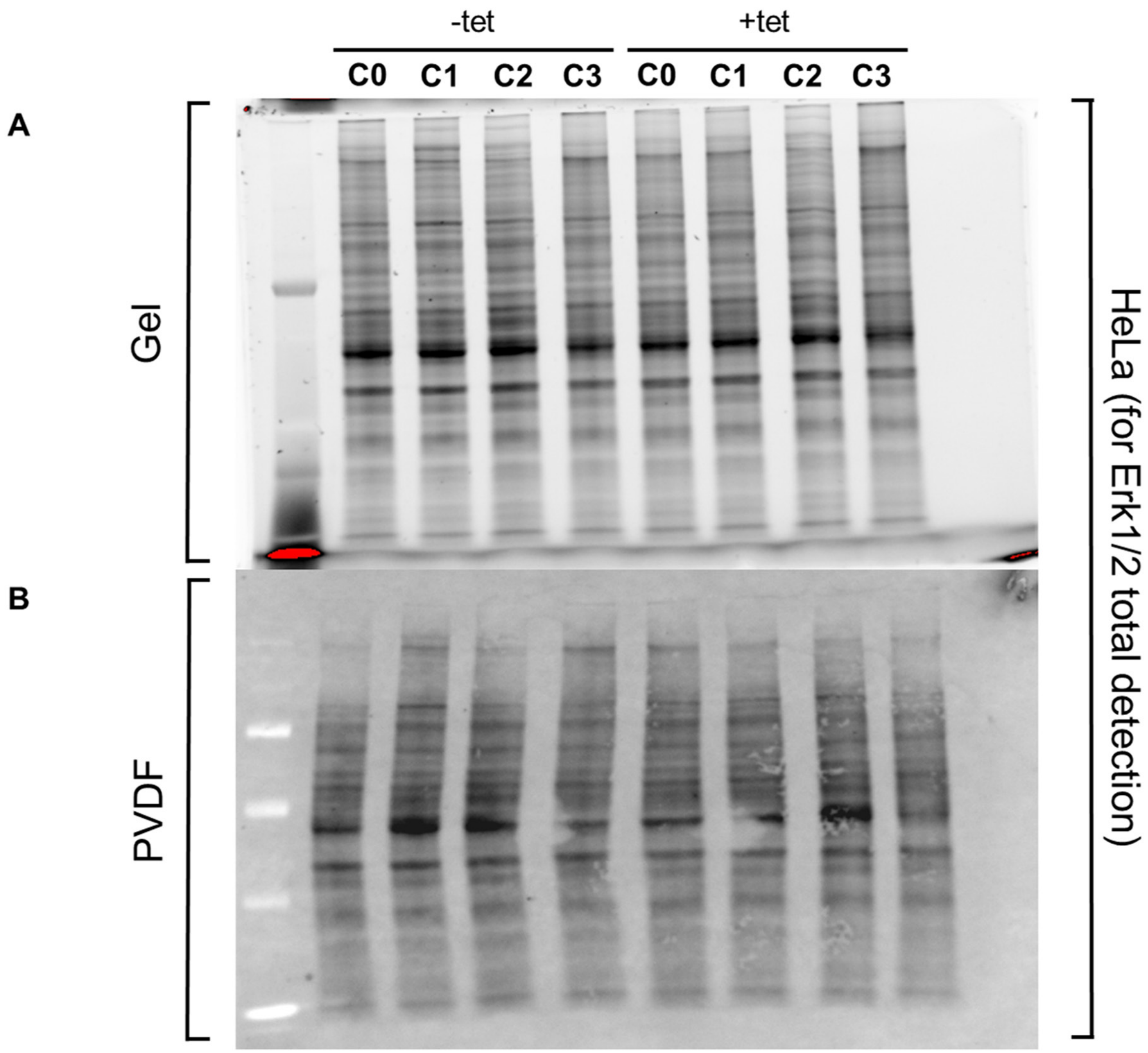


**Supplementary Figure 15: An example of the total protein staining of –tet and +tet HeLa cells.** Figure **(A)** depicts the gel with stained total protein. Figure **(B)** shows the total protein blotted from the gel onto the PVDF membrane. This membrane was used for further detection of total Erk1/2 shown in SF 8. Abbreviations: C, condition; Erk1/2, extracellular signal-regulated kinase 1/2; PVDF, polyvinylidene difluoride; tet, tetracycline; WB, Western blot.
